# ZnFe Layered Double Hydroxide Nanosheets Loaded with Cu Single‐Atom Nanozymes with Multi‐Enzyme‐Like Catalytic Activities as an Effective Treatment for Bacterial Keratitis

**DOI:** 10.1002/advs.202411999

**Published:** 2025-01-22

**Authors:** Keke Wang, Mao‐sen Yuan, Pengxiu Dai, Jing Li, Anju Tao, Xinke Zhang, Jinyi Wang, Qin Tu

**Affiliations:** ^1^ College of Chemistry and Pharmacy Northwest A&F University Yangling Shaanxi 712100 P. R. China; ^2^ College of Veterinary Medicine Northwest A&F University Yangling Shaanxi 712100 P. R. China

**Keywords:** antibacterial, bacterial keratitis, multi‐enzyme‐like activities, nanozyme, target‐oriented

## Abstract

Bacterial keratitis (BK) is a type of corneal inflammation resulting from bacterial infection in the eye. Although nanozymes have been explored as promising materials in corneal wound healing, currently available nanozymes lack sufficient catalytic activity and the ability to penetrate bacterial biofilms, limiting their efficacy against the treatment of BK. To remedy this, ZnFe layered double hydroxide (ZnFe‐LDH) nanosheets are loaded with Cu single‐atom nanozymes (Cu‐SAzymes) and aminated dextran (Dex‐NH_2_), resulting in the formation of the nanozyme DT‐ZnFe‐LDH@Cu, which possesses peroxidase (POD)‐, oxidase (OXD)‐, and catalase (CAT)‐like catalytic activities. This enables the nanozyme to generate reactive oxygen species (ROS), such as hydroxyl radicals (^•^OH), superoxide anion radical (O_2_^•−^), and singlet oxygen (^1^O_2_) from hydrogen peroxide (H_2_O_2_), thereby killing the bacteria causing the infections. The surface Dex‐NH_2_ enabled the DT‐ZnFe‐LDH@Cu to penetrate the biofilm and adsorb onto extracellular polymeric substances (EPS) produced by bacteria in the biofilm. Additionally, the DT‐ZnFe‐LDH@Cu successfully repaired *P. aeruginosa*‐infected corneal injury in a BK rabbit model more effectively than commercially available tobramycin eye drops. This was enabled, in part, by the ability of DT‐ZnFe‐LDH@Cu to reduce inflammation by promoting the polarization of pro‐inflammatory macrophages (M1) to anti‐inflammatory macrophages (M2) and decrease the expression of α‐smooth muscle actin (α‐SMA) to promote wound healing without scar formation. This study provides an innovative concept for the treatment of BK and holds great scientific value and clinical application potential.

## Graphical abstract

A targeted DT‐ZnFe‐LDH@Cu with multi‐enzyme‐like activities—POD‐, OXD‐, and CAT‐like activities is proposed, enabling the nanozyme to generate ROS, such as ^1^O_2_, O_2_^•−^ and ^•^OH, thereby killing the bacteria that cause the infection. The surface Dex‐NH_2_ enables DT‐ZnFe‐LDH@Cu to penetrate the biofilm and adsorb to extracellular polymer substances of bacteria within the biofilm, successfully repairing corneal damage in rabbits infected with *P. aeruginosa*.

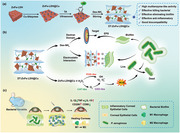

## 1 Introduction

Bacterial keratitis (BK), also known as corneal ulcers, is a potentially vision‐threatening eye infection that can ultimately lead to blindness.^[^ ^1^, ^2^, ^3^, ^4^ ^]^ Clinical treatment includes local administration—usually via eye drops or ointment—or intra‐corneal injection of antibiotics.^[^ ^5^, ^6^ ^]^ However, due to the unique anatomical, physiological, and structural characteristics of the cornea, such as the presence of tight intraepithelial junctions,^[^ ^7^, ^8^ ^]^ and the fact that the tear film dilutes administered drug solutions, causing the drug to be rapidly cleared from the surface of the cornea, the bioavailability of locally administered drugs is less than 5%.^[^ ^9^, ^10^, ^11^ ^]^ In addition, the overuse of antibiotics has led to the emergence of antibiotic‐resistant bacteria, clouding the future of antibiotic treatment.^[^ ^12^, ^13^, ^14^ ^]^ Our group previously reported the application of hydrogel‐supported nanoclusters to address these issues in the treatment of BK.^[^ ^15^ ^]^ However, external laser irradiation, long irradiation times, and high laser power may cause damage to the cornea.

In recent years, nanozymes—unique nanomaterials with catalytic properties that mimic those of enzymes—have been developed and applied in the treatment of BK because of their ease of preparation, long‐term and stable catalytic activity, and high barrier to resistance.^[^ ^16^, ^17^, ^18^, ^19^, ^20^ ^]^ However, their therapeutic efficacy is largely restricted by their poor, and often single, catalytic activities. Hence, enhancing the activity of nanozymes is a key challenge for improving their antibacterial effectiveness. Lamellar 2D nanosheets of layer double hydroxides (LDHs) with formulas of [M^2+^_1‐_ *_x_* M^3+^ *_x_* (OH)_2_] (A^n−^)*_x_*_/n_⋅mH_2_O represent promising scaffolds for the preparation of nanozymes.^[^ ^21^ ^]^ LDHs have a unique structure that endow the nanomaterials with high surface areas, anion exchange capabilities, catalytic properties, high biocompatibility, low toxicity, customable size and composition, and good stability.^[^ ^22^, ^23^, ^24^ ^]^ If LDHs is used as a carrier for the preparation of single‐atom nanozymes (SAEs), it not only has the high activity of single‐atom catalyst, but also inherits the advantages of LDHs, such as high biocompatibility, low toxicity, customable size and composition, and good stability. However, to our knowledge, there have been no reports of LDHs supporting SAEs to enhance the nanozyme activity for the treatment of BK.

While previously developed nanozymes have been employed to address drug‐resistant bacterial infections, cannot efficiently generate reactive oxygen species (ROS) in situ and lack the targeting of catalytically produced ROS because they typically boast only single enzyme‐like catalytic functions. In addition, their structures are not modified to adhere to target bacterial biofilms and adhere to the bacteria; instead, they inadvertently harm the surrounding normal cells and tissues as they diffuse from the release point to the bacteria.^[^ ^25^ ^]^ Second, during transport from the material to the site of infection, a fraction of the ROS generated by the nanozymes will be consumed by non‐bacterial components, thereby reducing the number ROS available for combating the bacteria.^[^ ^26^ ^]^ Third, bacteria can develop resistance mechanisms to adapt to the levels of ROS produced by conventional nanozymes, thereby reducing the bactericidal efficacy of the nanozymes.^[^ ^27^, ^28^ ^]^

**Scheme 1:**
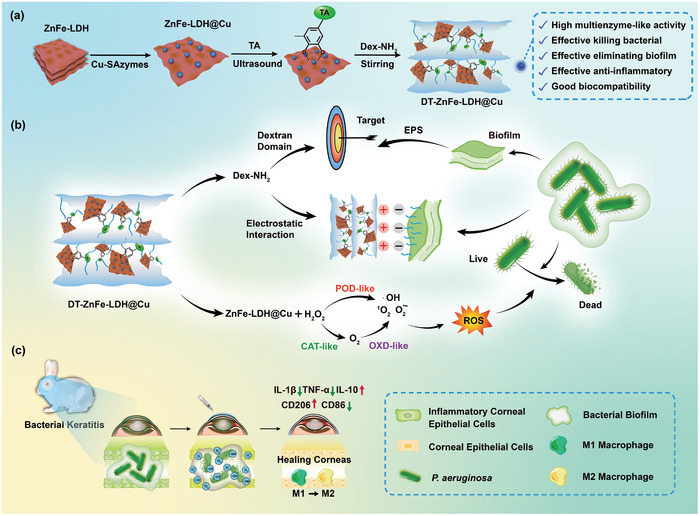
Schematic illustration of the preparation of DT‐ZnFe‐LDH@Cu and treatment strategy of BK. A) ZnFe‐LDH was loaded with Cu‐SAzymes, and then the TA crosslinker was combined with Dex‐NH_2_ to form DT‐ZnFe‐LDH@Cu. B) Bacteriostatic process diagram of DT‐ZnFe‐LDH@Cu. C) DT‐ZnFe‐LDH@Cu is injected into the bacterial cornea of rabbits, killing the bacteria and clearing the biofilm while concomitantly decreasing the expression of pro‐inflammatory factors and increasing the expression of anti‐inflammatory factors, thereby enabling the healing of the corneal tissue.

To address these issues, we constructed a biofilm‐targeted nanozyme with multi‐enzyme‐like activities to treat BK caused by bacterial infection. As shown in **Scheme** **1**a, ZnFe‐LDH nanosheets were employed as a carrier to support Cu single‐atom nanozymes (Cu‐SAzymes) to form a nanocatalyst (ZnFe‐LDH@Cu) with multi‐enzyme‐like catalytic activities. The ZnFe‐LDH@Cu was further modified with tannic acid (TA)^[^ ^29^, ^30^ ^]^ and aminated dextran (Dex‐NH_2_). The hydroxyl and carboxylic acid functional groups in TA form hydrogen bonds with Dex‐NH_2_ and coordinate metal cations in the ZnFe‐LDH@Cu structure, which we hypothesized would lead to the formation of a double cross‐linked Dex‐NH_2_‐TA‐ZnFe‐LDH@Cu nanozyme (DT‐ZnFe‐LDH@Cu). In addition, functionalization of the ZnFe‐LDH@Cu with Dex‐NH_2_—which was positively charged in solution—was intended to promote the binding of the nanocatalyst to extracellular polymeric substances (EPS) and the negatively charged surface of bacteria within the biofilm through electrostatic interactions, thereby facilitating penetration of the nanocatalyst into the biofilm to generate ROS and eradicate the bacteria in the biofilm.^[^ ^31^, ^32^, ^33^ ^]^

As expected, compared to Cu‐SAzymes alone, ZnFe‐LDH features a vertical structure with higher functional surface area, which helps to improve its stability as a carrier for Cu‐SAzymes, which provides more active sites for ZnFe‐LDH@Cu enabling multi‐enzyme‐like catalytic activities. During bacterial infection in the cornea, bacteria produce a variety of acidic metabolites, which lowers the pH of the wound environment to 4.5–6.5. The peroxidase (POD)‐ and oxidase (OXD)‐like activities of DT‐ZnFe‐LDH@Cu enable it to generate ROS—hydroxyl radicals (^•^OH), superoxide anion radical (O_2_^•−^), and singlet oxygen (^1^O_2_) —to kill bacteria in the biofilm within the wound. However, during the healing process as the biofilm shrinks, the physiological environment in the cornea becomes more neutral, during which the catalase (CAT)‐like activity of DT‐ZnFe‐LDH@Cu plays more of a primary role in converting excess hydrogen peroxide (H_2_O_2_) into oxygen (O_2_) to further alleviate the inflammatory response (Scheme 1b).^[^ ^34^, ^35^ ^]^ In vitro bacteriostatic experiment, DT‐ZnFe‐LDH@Cu inhibited the growth of *Pseudomonas aeruginosa* (*P. aeruginosa*) by more than 90%, indicating that it was capable of destroying the bacterial structure, and it demonstrated excellent biocompatibility and biosafety. In vivo, the therapeutic effect of DT‐ZnFe‐LDH@Cu on BK was superior to that of commercial tobramycin eye drops. Hematoxylin‐eosin (H&E) staining demonstrated that inflammatory factors did not infiltrate into the cornea of the DT‐ZnFe‐LDH@Cu treatment group, and the epidermis remained intact. Additionally, the results of immunohistochemical and immunofluorescence staining analyses indicated that the pro‐inflammatory factors tumor necrosis factor‐α (TNF‐α) and interleukin‐1β (IL‐1β) were down‐regulated, the anti‐inflammatory factor interleukin‐10 (IL‐10) was up‐regulated, the expression of CD86 decreased, and the expression of CD206 increased. This might be attributed to the ability of DT‐ZnFe‐LDH@Cu to promote the polarization of pro‐inflammatory M1 macrophages to anti‐inflammatory M2 macrophages, thereby reducing inflammation. Meanwhile, the expression levels of α‐smooth muscle actin (α‐SMA) were reduced, enabling better corneal recovery by preventing scaring (Scheme 1c). In summary, our work highlighted DT‐ZnFe‐LDH@Cu as a potential therapeutic for treating BK in the clinic and a novel platform for designing highly efficient catalytic and antimicrobial nanomaterials for utilization.

## 2 Results and Discussion

### 2.1 Synthesis and Characterization of ZnFe‐LDH@Cu

**Figure 1:**
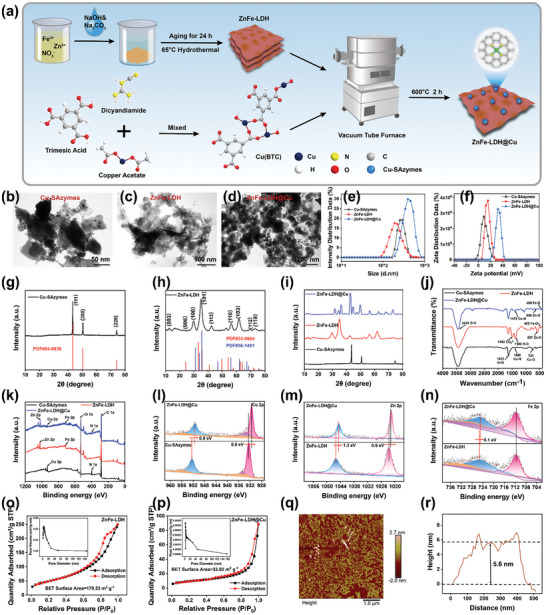
Synthesis and characterization of ZnFe‐LDH@Cu. a) Schematic illustration of the manufacturing process of ZnFe‐LDH@Cu. TEM images of Cu‐SAzymes b), ZnFe‐LDH c), and ZnFe‐LDH@Cu d). Particle size analysis diagram e) and Zeta point distribution map f) of Cu‐SAzymes, ZnFe‐LDH, and ZnFe‐LDH@Cu. g) XRD spectra of Cu‐SAzymes and the standard spectra of Cu_2_O (PDF#04‐0836). h) XRD spectra of ZnFe‐LDH and the standard spectra of ZnO (PDF#36‐1451) and Fe_3_O_4_ (PDF # 33‐0664). i–k) XRD spectra, FT‐IR spectra, and XPS full‐spectrum of Cu‐SAzymes, ZnFe‐LDH, and ZnFe‐LDH@Cu. l) High‐resolution Cu 2p XPS spectrum of Cu‐SAzymes and ZnFe‐LDH@Cu. High‐resolution XPS spectrum of Zn 2p m) and Fe 2p n) in ZnFe‐LDH and ZnFe‐LDH@Cu. N_2_ adsorption–desorption isotherms and pore size distribution (illustration) of ZnFe‐LDH o) and ZnFe‐LDH@Cu p). q) AFM image of ZnFe‐LDH@Cu. r) The thickness distribution of ZnFe‐LDH@Cu as measured by the AFM image is shown in the white line.

First, ZnFe‐LDH nanosheet arrays were synthesized by a co‐precipitation method, and separately, ultra‐thin 2D nanosheets of Cu‐SAzymes were produced through a dicyandiamide‐assisted pyrolysis strategy.^[^ ^36^ ^]^ Ultrahigh‐resolution field emission transmission electron microscopy (URFE‐TEM) analysis confirmed the anchoring of copper single atoms on the nitrogen‐doped carbon matrix support (marked with a red circle) (Figure S1, Supporting Information). Subsequently, ZnFe‐LDH was introduced into the Cu‐SAzymes precursor solution and agitated for 5 h, followed by reduction with H_2_/Ar at 600 °C for 2 h, resulting in the formation of ZnFe‐LDH@Cu (**Figure** **1**a). The morphologies of the synthesized materials—Cu‐SAzymes, ZnFe‐LDH, and ZnFe‐LDH@Cu—were characterized by transmission electron microscopy (TEM) as depicted in Figure 1b–d. Cu‐SAzymes featured a graphene‐like nanosheet morphology, while the TEM images of ZnFe‐LDH nanosheets revealed a layered “nanosheet on nanosheet” configuration, indicative of a complete vertical structure and a multilayered ZnFe‐LDH architecture. This “nanosheet‐on‐nanosheet” sandwich structure not only provided an increased surface area and additional catalytic active sites for redox reactions but also ensured high loading efficiency. Figure 1d illustrates the thin and well‐dispersed nature of ZnFe‐LDH@Cu, with Cu‐SAzymes firmly adhering to the surface of ZnFe‐LDH.

Dynamic light scattering (DLS) measurements determined the hydrodynamic diameters of Cu‐SAzymes and ZnFe‐LDH to be ≈275 and 201 nm, respectively, while the diameter of ZnFe‐LDH@Cu was ≈397 nm. This increase in diameter indicated the successful incorporation of the Cu‐SAzymes onto ZnFe‐LDH (Figure 1e). Dispersibility experiments demonstrated that ZnFe‐LDH@Cu exhibited high dispersion in water for up to 12 h (Figure S2, Supporting Information). The zeta potentials for Cu‐SAzymes, ZnFe‐LDH, and ZnFe‐LDH@Cu were measured at 8.92, 15.53, and 32.06 mV, respectively (Figure 1f), suggesting that the increase in zeta potential for ZnFe‐LDH@Cu was indicative of successful loading of the Cu‐SAzymes onto ZnFe‐LDH. The X‐ray diffraction (XRD) pattern of the Cu‐SAzymes (Figure 1g) featured peaks consistent with Cu_2_O (PDF # 04‐0836). The XRD pattern for the synthesized ZnFe‐LDH featured characteristic peaks at 2*θ* = 12.6° and 24.3°, which correspond to the (003) and (006) planes of the LDH phase, respectively, while the peaks at 56.2° and 61.8° correspond to the (110) and (113) planes of the LDH phase (Figure 1h). The additional peaks at 31.0°, 33.9°, 62.1°, 68.4°, and 71.9° are attributed to the (100), (101), (103), (112), and (119) planes, respectively, aligning with the characteristic lines of ZnO (PDF # 36‐1451) and Fe_3_O_4_ (PDF # 33‐0664). The characteristic bands of Cu‐SAzymes and ZnFe‐LDH were also evident in the XRD pattern of ZnFe‐LDH@Cu (Figure 1i), confirming the successful synthesis of the composite material ZnFe‐LDH@Cu.

Figure 1j presents the Fourier transform infrared spectroscopy (FT‐IR) spectra of Cu‐SAzymes, ZnFe‐LDH, and ZnFe‐LDH@Cu. The peaks at 1622, 1420, and 724 cm^−1^ in the FT‐IR of Cu‐SAzymes were attributed to the stretching vibration of C≐O, Cu‐N, and Cu‐O, respectively. The FT‐IR spectrum of ZnFe‐LDH exhibited distinguishing peaks at 3420 cm^−1^, corresponding to the stretching mode of the ‐OH group associated with water molecules in the LDH layer. The absorption band at 1482 cm^−1^ represented the symmetric and asymmetric stretching modes of CO_3_^2−^ inserted between LDH layers, while the peaks at 607 and 485 cm^−1^ were attributed to the lattice vibrations (Zn‐O and Fe‐O) within the LDH structure. The FT‐IR spectrum of ZnFe‐LDH@Cu contained characteristic peaks of 3420 cm^−1^ (O‐H tensile vibration), 1478 cm^−1^ (CO_3_^2−^ tensile vibration), 658 cm^−1^ (Zn‐O tensile vibration), and 460 cm^−1^ (Fe‐O tensile vibration), confirming the successful incorporation of Cu‐SAzymes into the ZnFe‐LDH. In addition, the surface chemical states of Cu‐SAzymes, ZnFe‐LDH, and ZnFe‐LDH@Cu were investigated using X‐ray photoelectron spectroscopy (XPS) (Figure 1k). The full‐size XPS indicated the presence of Zn, Fe, and Cu elements in the ZnFe‐LDH@Cu. The high‐resolution Cu 2p spectrum of Cu‐SAzymes featured peaks at 952.1 and 932.2 eV, corresponding to Cu^+^, and peaks at 956 and 932.8 eV matched Cu^2+^ (Figure 1l). This suggested that Cu single atoms could serve as REDOX sites required to simulate enzyme catalysis. Compared to ZnFe‐LDH@Cu, the Cu 2p binding energy moved to a lower direction, indicating that Cu‐SAzymes loading on ZnFe‐LDH changed the central electronic structure of Cu^+^ and Cu^2+^. In the Zn 2p spectrum of ZnFe‐LDH@Cu, the peaks at 1021.3 and 1044.9 eV were identified as Zn 2p_3/2_ and Zn 2p_1/2_, respectively (Figure 1m). Lastly, the Fe 2p spectrum of ZnFe‐LDH@Cu revealed peaks at 711.1 and 728.2 eV, which were assigned to Fe^2+^, while the peaks at 917.8 and 725.4 eV correspond to Fe^3+^ (Figure 1n). Compared with undoped Cu‐SAzymes, the spin‐orbit peaks of Zn and Fe in the ZnFe‐LDH were shifted toward lower binding energies, indicating that the addition of Cu‐SAzymes also impacted on the central electronic structure of Zn and Fe in the ZnFe‐LDH.

Moreover, the load of Cu atom determined by inductively coupled plasma atomic emission spectroscopy (ICP‐OES) was 7.05 wt.% (Table S1, Supporting Information). The specific surface area and pore structure of ZnFe‐LDH and ZnFe‐LDH@Cu were determined by N_2_ adsorption‐desorption experiments. The type *iv* isotherm indicated the existence of mesoporous structures, as illustrated in Figure 1o (illustrated as pore size distribution curve). The BET surface area of ZnFe‐LDH was 179.53 m^2^·g^−1^, with an average pore diameter of ≈3.3 nm, while the BET surface area of ZnFe‐LDH@Cu was 33.82 m^2^·g^−1^, with an average pore diameter of ≈2.8 nm (Figure 1p). The decrease in BET surface area and pore diameter also indicated the successful incorporation of Cu‐SAzymes. Moreover, although there was a decrease in specific surface area, the catalytic activity remained unaffected, as the oxidation state of the Cu ions in the Cu‐SAzymes was primarily Cu^+^, which aggregated stably within the ZnFe‐LDH structure. This configuration provided more reactive active sites, as demonstrated in subsequent multi‐enzyme‐like activities experiments. The thickness of ZnFe‐LDH@Cu was measured by atomic force microscopy (AFM) (Figure 1q), the results of which revealed a thickness of ≈5.6 nm (Figure 1r), thereby confirming the successful preparation of the ultra‐thin ZnFe‐LDH@Cu (Figure S3, Supporting Information). All these observations confirmed the successful fabrication of the ZnFe‐LDH@Cu.

### 2.2 Synthesis and Characterization of DT‐ZnFe‐LDH@Cu

**Figure 2:**
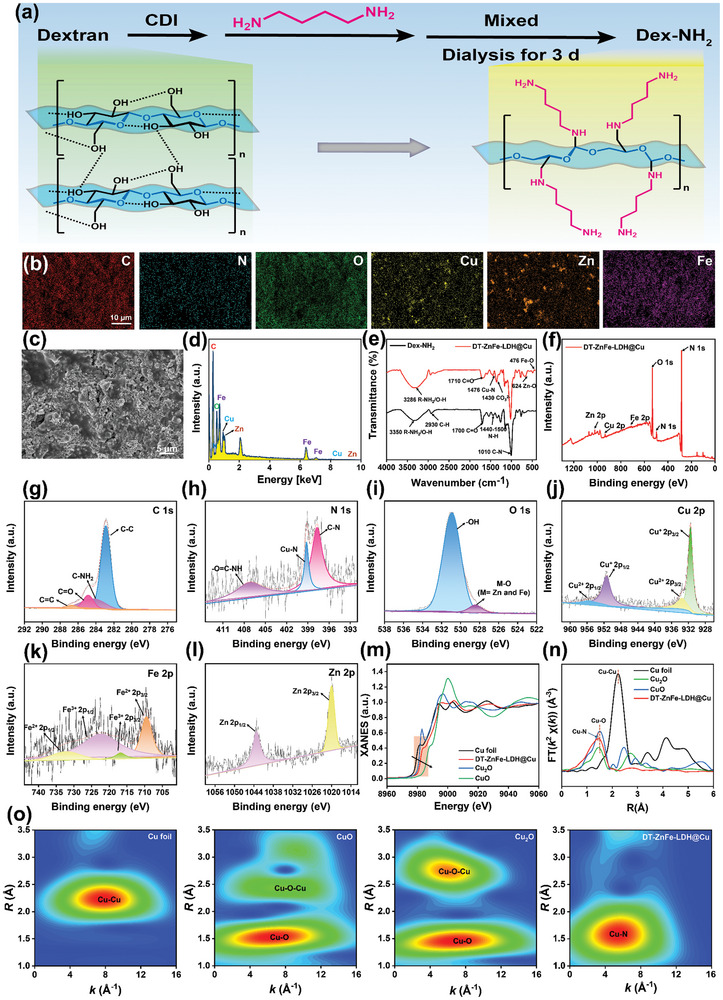
Synthesis and characterization of DT‐ZnFe‐LDH@Cu. a) Diagram of the synthesis process of Dex‐NH_2_. b) Corresponding elemental mapping of DT‐ZnFe‐LDH@Cu. c) SEM image of DT‐ZnFe‐LDH@Cu. d) EDS spectrum of DT‐ZnFe‐LDH@Cu. e) FT‐IR spectra of Dex‐NH_2_ and DT‐ZnFe‐LDH@Cu. f) XPS full‐spectrum of DT‐ZnFe‐LDH@Cu. High‐resolution XPS spectra of C 1s g), N 1s h), O 1s i), Cu 2p j), Zn 2p k), and Fe 2p l) in DT‐ZnFe‐LDH@Cu. m) Cu K‐edge normalized XANES spectra of DT‐ZnFe‐LDH@Cu with reference materials Cu foil, Cu_2_O, and CuO. n) Fourier transform at the Cu K‐edge of DT‐ZnFe‐LDH@Cu and reference samples. o) WT of Cu foil, CuO, Cu_2_O, and DT‐ZnFe‐LDH@Cu.

LDHs boasted a high aspect ratio and specific surface area, possessed an inherent van der Waals gap between their hierarchical structures, which facilitated the insertion of different guest species. To endow the nanozymes with the ability to target and infiltrate into bacterial biofilms, Dex‐NH_2_ ligands were introduced into the ZnFe‐LDH@Cu structure. We believed these positively charged ligands would facilitate binding of the nanomaterial to bacterial cell membranes and EPS within the biofilm, promoting deeper penetration into the biofilm and enhancing sterilization efficacy. The synthesis process and the typical proton nuclear magnetic resonance (^1^H NMR) spectrum of Dex‐NH_2_ are shown in **Figures** **2**a and S4 (Supporting Information), respectively. In the ^1^H NMR spectrum, the signal at δ = 4.68 ppm was associated with the methylidyne protons (a, O‐CH‐O) of glucose units of dextran. The signals at δ = 2.67 ppm correspond to the methylene protons adjacent amine groups (b, CH_2_‐NH_2_). Based on the area ratio of peak a (corresponding to the amount of total glucose units of Dex‐NH_2_) and peak b (corresponding to twice the amount of aminated glucose units of Dex‐NH_2_), it was calculated that ≈1.6 {0.80/(0.47/2)} glucose units of Dex‐NH_2_ possess one amino group, confirming the successful coupling of the amino group was to the glucose unit of dextran. TA, a polyphenol structure substance, was employed as a crosslinking agent, forming coordination bonds with Fe ions in ZnFe‐LDH. Additionally, it facilitated the formation of hydrogen bonds with Dex‐NH_2_, resulting in the composite DT‐ZnFe‐LDH@Cu. The microstructure of DT‐ZnFe‐LDH@Cu after freeze‐drying was observed by scanning electron microscopy (SEM) (Figure 2c). After chemical grafting of Dex‐NH_2_ and TA on the surface ZnFe‐LDH@Cu, a large number of nanostructures were observed on the surface of the combined structure, indicating successful synthesis of DT‐ZnFe‐LDH@Cu. The element composition of DT‐ZnFe‐LDH@Cu was analyzed through the element mapping of DT‐ZnFe‐LDH@Cu (Figure 2b), revealing a homogeneous distribution of Zn, Fe, and Cu within the DT‐ZnFe‐LDH@Cu. This elemental distribution was further corroborated by energy‐dispersive X‐ray spectroscopy (EDS) (Figure 2d).

FT‐IR was employed to characterize DT‐ZnFe‐LDH@Cu (Figure 2e). A prominent absorption peak for the carbonyl group (C≐O) at 1700 cm^−1^ was identified in Dex‐NH_2_, indicating the successful synthesis of amino‐modified dextran. Compared with Dex‐NH_2_, DT‐ZnFe‐LDH@Cu exhibited new peaks at ≈1476, ≈1430, ≈624, and ≈476 cm^−1^, which correspond to the presence of ‐OH, ‐Cu‐N, CO_3_^2−^ and M‐OH (M = Zn or Fe), respectively. Following grafting of TA, the hydroxyl group of phenol was found to interact with Fe ion in ZnFe‐LDH@Cu to form a dynamic coordination bond that contributed to the attenuation of the ‐OH/‐NH absorption peak. Additionally, the presence of enhanced peaks for C‐O, CO_3_^2−^, and M‐OH (M = Zn or Fe) peaks indicated the in situ growth of ZnFe‐LDH@Cu and successful connection of TA in the Dex‐NH_2_.

Additionally, the surface composition and electron states of DT‐ZnFe‐LDH@Cu were investigated by XPS. The XPS spectra confirmed the existence of Zn, Fe, Cu, C, N, and O elements (Figure 2f). In the high‐resolution XPS map of C 1s (Figure 2g), peaks at 286.68, 284.88, 284.38, and 282.88 eV were observed, corresponding to C≐C, C≐O, C‐NH_2_, and C─C bonds, respectively. Figure 2h displayed the high‐resolution XPS spectrum of N 1s, which featured peaks at 407.08, 399.18, and 397.68 eV, corrresponding to O≐C‐NH_2_, Cu‐N, and C─N bonds, respectively. These findings further corroborated the successful synthesis of Dex‐NH_2_ and the effective incorporation of Cu‐SAzymes. The high‐resolution O 1s spectrum (Figure 2i) featured two peaks of ≈ 530.88 and ≈ 528.48 eV, which, upon fitting, correspond to ‐OH and M‐O (M = Zn, Fe), respectively, indicating a significant presence of ‐OH from TA and the presence of ZnFe‐LDH. The Cu 2p spectrum featured by two main peaks, Cu 2p_1/2_ (951.38 eV) and Cu 2p_3/2_ (931.48 eV), indicating that the majority of the Cu in the material was present as Cu^+^ in a high‐spin state (Figure 2j). The two satellite bands at 955.58 and 933.58 eV both corresponded to a Cu^2+^ speciation. This characteristic allowed Cu‐SAzymes to function as the requisite REDOX site in simulating enzyme catalysis. The high‐resolution XPS spectra of Zn 2p are shown in Figure 2l. The peaks at 1019.98 and 1043.18 eV, representing Zn 2p_3/2_ and Zn 2p_1/2_, respectively. In the Fe 2p spectrum (Figure 2k), the two main peaks at 709.48 and 722.38 eV belong to Fe 2p_3/2_ and Fe 2p_1/2_, respectively, and the peaks of Fe 2p_3/2_ were fitted to two peaks centered on 709.48 and 716.78 eV, which were associated with Fe^2+^ and Fe^3+^, respectively. The above resulted indicated the successful synthesis of DT‐ZnFe‐LDH@Cu.

To disclose the coordination environment and chemical state of Cu sites in the DT‐ZnFe‐LDH@Cu, the X‐ray absorption near‐edge structure (XANES) and extended X‐ray absorption fine structure (EXAFS) spectroscopy were investigated. As shown in Figure 2m, the Cu K‐edge XANES spectrum of DT‐ZnFe‐LDH@Cu exhibited an energy edge distribution between Cu_2_O and CuO, suggested that the oxidation state of copper atoms in DT‐ZnFe‐LDH@Cu was higher than 1^+^ but lower than 2^+^, which was consistent with the Cu high‐resolution XPS result in Figure 2j. The coordination structure of Cu atoms was further investigated by Fourier‐transformed (FT) k^3^‐weighted EXAFS spectrum, there was only one main peak at 1.44 Å, which was assigned to the first coordination shell of Cu‐N scattering path (Figure 2n), consistent with the high‐resolution XPS of N 1s containing a Cu‐N coordination peak (Figure 2h). Importantly, compared with Cu foil, no measurable metallic Cu‐Cu scattering peak ≈2.20 Å was found for DT‐ZnFe‐LDH@Cu, manifesting that Cu exists as an isolated atom dispersed on the DT‐ZnFe‐LDH@Cu. Cu atoms were anchored in a plane via 3.66 N atoms of 1.94 Å based on the quantitative EXAFS fitting analysis in k and R space (Figure S5a,b and Table S2, Supporting Information). Moreover, the wavelet transform (WT) of the Cu K‐edge EXAFS oscillations of DT‐ZnFe‐LDH@Cu was carried out, and the WT maximum at 4.65 Å^−1^ could be assigned to the Cu‐N scattering (Figure 2o). The reference Cu foil demonstrated an intensity maximum at ≈7.76 Å^−1^ and ≈2.23 Å, with no such Cu‐Cu bonding feature being observed for DT‐ZnFe‐LDH@Cu. Consequently, based on the URFE‐TEM, XPS, and X‐ray absorption spectroscopy (XAS) results, it was strongly confirmed that Cu was atomically dispersed.

### 2.3 Multi‐Enzyme‐Like Activity of ZnFe‐LDH@Cu

**Figure 3:**
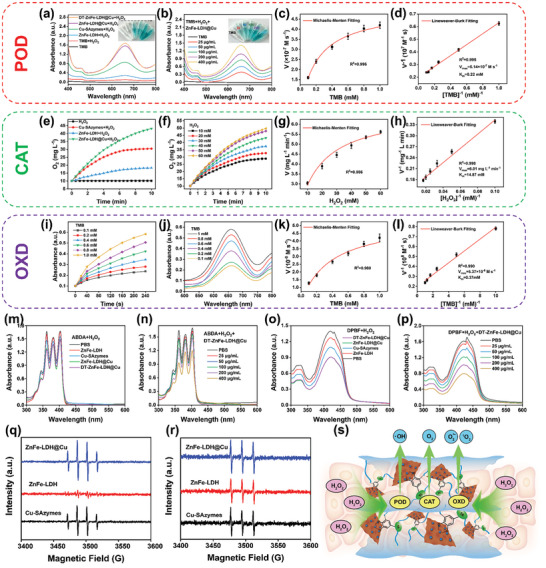
Characterizations of multi‐enzymatic properties of DT‐ZnFe‐LDH@Cu. a) UV–vis spectra of oxTMB by POD‐like activity produced by different components. The inset image is the corresponding color change. b) Absorbance curves of oxTMB after incubating with varying concentrations of DT‐ZnFe‐LDH@Cu. The inset image is the corresponding color change. c) Michaelis–Menten kinetic analysis and d) Lineweaver‐Burk plots of ZnFe‐LDH@Cu with TMB as substrates. e) Analysis of dissolved oxygen concentration of different component solutions with time extension. f) Dissolved oxygen concentration analysis of ZnFe‐LDH@Cu solution under different concentrations of H_2_O_2_ with time extension. g) Michaelis–Menten kinetic analysis and h) Lineweaver‐Burk plots of ZnFe‐LDH@Cu with H_2_O_2_ as substrates. i) Absorbance changes of ZnFe‐LDH@Cu with time extension under different concentrations of TMB. j) UV–vis spectra of ZnFe‐LDH@Cu oxTMB at different concentrations. k) Michaelis–Menten kinetic analysis and l) Lineweaver‐Burk plots of ZnFe‐LDH@Cu with TMB as substrates. UV–vis spectra of the catalyzed oxidation of ABDA and DPBF in different components m,o) and concentrations conditions n,p). ESR spectra for detection of ⋅OH by DMPO q) and ^1^O_2_ by TEMP r) in Cu‐SAzymes, ZnFe‐LDH, and ZnFe‐LDH@Cu. s) Schematic illustration of the multienzyme‐like of DT‐ZnFe‐LDH@Cu.

Both Cu‐SAzymes and ZnFe‐LDH have previously demonstrated various enzyme‐like catalytic activities, such as POD‐, CAT‐, and OXD‐like activities, albeit individually.^[^ ^37^, ^38^, ^39^ ^]^ Thus, we envisaged that the combination of the two components would endow the ZnFe‐LDH@Cu with multi‐enzyme‐like activities. We first investigated the POD‐like activity of ZnFe‐LDH@Cu (100 µg mL^−1^), which would enable the material to catalyze the decomposition of H_2_O_2_ into ^•^OH in acidic media. To assess the POD‐like activity of ZnFe‐LDH@Cu, we performed colorimetric assays using 3,3′,5,5′‐tetramethylbenzidine (TMB) to measure the changes in the absorbance at 652 nm. Upon production of ^•^OH, TMB was oxidized to form the blue‐colored TMB (oxTMB), which featured a maximum absorbance at 652 nm. As shown in **Figure** **3**a, there was no change in the Ultraviolet (UV) absorption peaks of the TMB alone and TMB + H_2_O_2_ groups. Upon the addition of ZnFe‐LDH to the reaction solution, the intensity of the absorption maximum at 652 nm belonging to oxTMB increased only slightly compared to the TMB alone and TMB + H_2_O_2_ groups, suggesting a poor POD‐like activity of ZnFe‐LDH. In contrast, once ZnFe‐LDH@Cu was added, the signal intensity at 652 nm increased sharply, even more significantly than the Cu‐SAzymes, indicating that the ZnFe‐LDH@Cu possessed a superior POD‐like activity. Moreover, it was found that the intensity of the absorption peak at 652 nm did not decrease after the addition of DT‐ZnFe‐LDH@Cu, which indicated that Dex‐NH_2_ and TA had no effect on the enzyme‐like activity of ZnFe‐LDH@Cu. As shown in the image in the inset of Figure 3a, there was no color change in the control group, while a change from colorless to blue was observed in the ZnFe‐LDH@Cu group. Next, we explored the dependence of the concentration of ZnFe‐LDH@Cu on the catalytic oxidation of TMB. As shown in Figure 3b, as the concentration of ZnFe‐LDH@Cu increased from 25 to 400 µg mL^−1^, the intensity of the absorption peak of TMB at 652 nm increased from 0.38 to 1.41 a.u., indicating that the POD‐like activity of ZnFe‐LDH@Cu was concentration‐dependent. We further investigated the kinetics of the POD‐like activity of ZnFe‐LDH@Cu in the presence of TMB and H_2_O_2_ as substrates with varying concentrations according to Michaelis–Menten kinetics. The initial velocity was determined by measuring the slope of the initial linear range of the reaction curve (Figure S6, Supporting Information). A Michaelis–Menten curve was produced by plotting the calculated initial velocity (*V*) against the TMB and H_2_O_2_ concentrations (Figure 3c). In the presence of H_2_O_2_, the maximum reaction velocity (*V*_max,_ a measure of the catalytic activity of ZnFe‐LDH@Cu) and Michaelis‐Menten constant (*K*_m_, a measure of the affinity of the nanozyme for the substrate) of ZnFe‐LDH@Cu were determined to be 23.13 × 10^−8^ m·s^−1^ and 32.75 mm, respectively (Figure S6, Supporting Information), while the corresponding parameters of ZnFe‐LDH@Cu in the presence of TMB as a substrate were 51.4 × 10^−8^ m·s^−1^ and 0.22 mm, respectively (Figure 3d).

In addition, we assessed the CAT‐like activity of ZnFe‐LDH@Cu (100 µg mL^−1^) using the dissolved O_2_ method. As shown in Figure 3e, no O_2_ was produced in the presence of H_2_O_2_ alone. However, Cu‐SAzymes, ZnFe‐LDH, and ZnFe‐LDH@Cu all catalyzed the conversion of H_2_O_2_ into O_2_, as evidenced by the gradual increase in the concentration of O_2_ measured as a function of time. Within 10 min, the dissolved O_2_ concentration detected in the solution containing ZnFe‐LDH@Cu + H_2_O_2_ was higher than in the solutions containing Cu‐SAzymes + H_2_O_2_ and ZnFe‐LDH + H_2_O_2_, which confirmed the superior CAT‐like activity of ZnFe‐LDH@Cu. We also explored the effect of H_2_O_2_ concentration on the catalytic ability of ZnFe‐LDH@Cu (Figure 3f). As the concentration of H_2_O_2_ increased from 10 to 60 mm, the concentration of dissolved oxygen increased from 28.52 to 49.94 mg·L^−1^. Applying the Michaelis–Menten equation to the kinetics data, the resulting enzymatic parameters *K*_m_ and *V*_max_ of ZnFe‐LDH@Cu were calculated to be 14.87 mm and 6.01 mg·L^−1^·min^−1^, respectively (Figure 3g,h).

Lastly, we explored the OXD‐like catalytic activity of ZnFe‐LDH@Cu by measuring the kinetics of conversion of O_2_ to O_2_^•−^ and ^1^O_2_ in the presence of H_2_O_2_ using UV–vis spectroscopy‐based TMB assays. As shown in Figure 3i,j, we first explored the dependence of ZnFe‐LDH@Cu OXD‐like activity on TMB substrate concentration. As the concentration of TMB increased from 0.1 to 1.0 mm, the intensity of the absorption maximum at 652 nm increased 2.42‐fold, corroborating the ability of ZnFe‐LDH@Cu to oxidize TMB to oxTMB in the presence of H_2_O_2_. When we performed steady‐state dynamics at a fixed ZnFe‐LDH@Cu concentration (100 µg mL^−1^) and varied the TMB concentration from 0.1 to 1.0 mm, the resulting plot of the *V* versus concentration of TMB demonstrated classical Michaelis–Menten dynamics (Figure 3k). The corresponding Lineweaver–Burk diagram (Figure 3l) was then used to calculate the kinetic parameters (i.e., *V*_max_ and *K*_m_) of the ZnFe‐LDH@Cu nanozyme, from which the *V*_max_ and K_m_ of ZnFe‐LDH@Cu were calculated to be 5.37 × 10^−8^ M·s^−1^ and 0.37 mm, respectively.

To further verify the generation of ^•^OH and ^1^O_2_, we supplemented each reaction solution with 9,10‐anthracenediyl‐bis(methylene)dimalonic acid (ABDA, a ^1^O_2_ probe), and measured the change in absorbance at 420 nm by UV–vis spectroscopy. As shown in Figure 3m, there was a negligible difference in the intensity of the absorption peak of the ZnFe‐LDH and Cu‐SAzymes solution at 420 nm compared to PBS. Although, the peak intensities of the DT‐ZnFe‐LDH@Cu‐ and ZnFe‐LDH@Cu‐containing reaction solutions were significantly lower compared to the ZnFe‐LDH or Cu‐SAzymes, there was no significant difference between DT‐ZnFe‐LDH@Cu and ZnFe‐LDH@Cu. Furthermore, when the concentration of DT‐ZnFe‐LDH@Cu (25, 50, 100, 200, and 400 µg mL^−1^) changed, the degree of oxidation also changed in a concentration‐dependent manner (Figure 3n). The OXD‐like activity of DT‐ZnFe‐LDH@Cu for converting O_2_ to O_2_^•−^ and ^1^O_2_ was also further evaluated using 1,3‐diphenylisobenzofuran (DPBF).^[^ ^36^, ^40^ ^]^ The intensity of the absorbance maximum at 420 nm of the DT‐ZnFe‐LDH@Cu and ZnFe‐LDH@Cu containing solutions decreased to the greatest degree relative to PBS, which was in great contrast with that of the ZnFe‐LDH and Cu‐SAzymes, indicating that they had obvious OXD‐like activity (Figure 3o). Similarly, in the presence DPBF and H_2_O_2_, when the concentration of DT‐ZnFe‐LDH@Cu varied (25, 50, 100, 200, and 400 µg mL^−1^), the absorbance of DPBF at 420 nm decreased in a concentration‐dependent manner (Figure 3p). This suggested that DT‐ZnFe‐LDH@Cu had higher OXD‐like activity than ZnFe‐LDH or Cu‐SAzymes alone, and its OXD‐like activity was concentration‐dependent.

Electron spin resonance (ESR) spectroscopy was used to detect the formation of short‐lived ROS radicals produced in the ZnFe‐LDH@Cu catalytic reaction, with 5,5‐dimethyl‐1‐pyrroline *N*‐oxide (DMPO) being utilized as a spin‐trapping agent to identify the formation of ^•^OH. As shown in the EPR spectra in Figure 3q, a quartet with a 1:2:2:1 ratio corresponding to the formation of a DMPO/^•^OH adduct was observed with all three materials tested—ZnFe‐LDH@Cu, ZnFe‐LDH, and Cu‐SAzymes. However, the intensity of the quartet in the EPR spectrum of ZnFe‐LDH@Cu was significantly higher than in the spectrum of ZnFe‐LDH, and slightly higher than that of Cu‐SAzymes, indicating that ZnFe‐LDH@Cu had POD‐like activity and catalyzed the production of ^•^OH. In addition, 2,2,6,6‐tetramethylpiperidine (TEMP) was used as a ^1^O_2_ trapping agent. TEMP reacted with ^1^O_2_, forming a triplet with a ratio of 1:1:1 (Figure 3r). The intensity of the triplet generated in the solution containing ZnFe‐LDH@Cu was the strongest of three materials, indicating that the ZnFe‐LDH@Cu generated the highest amount of ^1^O_2_.

When comparing DT‐ZnFe‐LDH@Cu to other nanozymes reported in the literature, POD‐ and CAT‐like activities of DT‐ZnFe‐LDH@Cu were medium to high, and OXD‐like activity was medium (Tables S3–S5, Supporting Information). The aforementioned experimental results validate the superior catalytic efficiency of ZnFe‐LDH@Cu compared to the Cu‐SAzymes and ZnFe‐LDH alone, which was mainly ascribed to the structural modulation of the nanocatalyst. The vertical structure of the lamellar ZnFe‐LDH nanosheets increased the functional surface area to maximize the number of reactions between the ZnFe‐LDH@Cu and H_2_O_2_ relative to the Cu‐SAzymes. Furthermore, the layering of the Cu‐SAzymes on the ZnFe‐LDH provided additional structural stability to the ZnFe‐LDH. The combination of the ZnFe‐LDH and Cu‐SAzymes also endowed the material with multi‐enzyme‐like catalytic activities—POD‐, CAT‐, and OXD‐like activities—distinguishing it from most nanozymes, which possessed only one or two enzyme‐like catalytic activities (Figure 3s).

### 2.4 In Vitro Antibacterial and Antibiofilm Properties of DT‐ZnFe‐LDH@Cu

**Figure 4:**
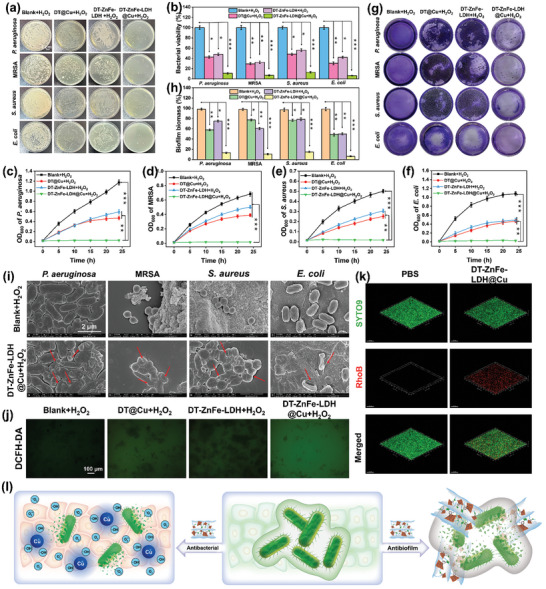
Antibacterial and antibiofilm activity of DT‐ZnFe‐LDH@Cu in vitro. a) The agar plate experiment and b) the corresponding quantitative results of *P. aeruginosa*, MRSA*, S. aureus*, and *E. coli* bacterial survival after different treatments. The OD_600_ of *P. aeruginosa* c), MRSA d)*, S. aureus* e), and *E. coli* f) under different treatments. g) Crystal violet staining of biofilms and h) the corresponding quantitative results of various bacteria after different treatments. i) SEM visualizing morphology of bacteria incubated with different treatment groups. j) ROS levels in *P. aeruginosa* was monitored by DCFH‐DA after different treatments. k) 3D CLSM images of *P. aeruginosa* biofilm were processed by RhoB‐labeled DT‐ZnFe‐LDH@Cu. Green: live bacteria, red: RhoB‐labeled DT‐ZnFe‐LDH@Cu. l) Schematic illustration of the antibacterial and antibiofilm effect of DT‐ZnFe‐LDH@Cu. Data are presented as mean ± SD (*n* = 3). (*p < 0.05, **p < 0.01, and ***p < 0.001).

The unique, multi‐enzyme‐like catalytic activities of ZnFe‐LDH@Cu in DT‐ZnFe‐LDH@Cu prompted us to comprehensively explore its antibacterial and antibiofilm potency against four species of commonly studied bacteria—*P. aeruginosa*, methicillin‐resistant *Staphylococcus aureus* (MRSA), *S. aureus*, and *E. coli*. The antibacterial properties of DT@Cu, DT‐ZnFe‐LDH, and DT‐ZnFe‐LDH@Cu were evaluated qualitatively and quantitatively by the standard colony‐forming unit (CFU) counting method on agar plates (**Figure** **4**a,b). In the Blank + H_2_O_2_ treatment group, there was a large number of bacterial colonies that formed following incubation, indicating that the concentration of H_2_O_2_ alone that was added was not high enough to inhibit bacterial growth. Exposure of all four bacteria to DT@Cu and DT‐ZnFe‐LDH resulted in slight inhibition of growth, indicating that DT@Cu and DT‐ZnFe‐LDH could not efficiently catalyze the conversion of H_2_O_2_ to ^•^OH to destroy bacteria. On the contrary, the bactericidal activity of DT‐ZnFe‐LDH@Cu was over 85%, which confirmed that loading the Cu‐SAzymes onto the ZnFe‐LDH endowed the ZnFe‐LDH@Cu with multi‐enzyme‐like activities and, consequently, more potent bactericidal properties. These results were further confirmed when we measured the growth curves of the four species of bacteria in the presence of each nanomaterial over a period of 24 h by measuring the changes in OD_600_. In the Blank + H_2_O_2_ group, all four bacteria grew linearly, indicating that the H_2_O_2_ had little‐to‐no effect on bacterial growth. When treated with DT@Cu + H_2_O_2_ and DT‐ZnFe‐LDH + H_2_O_2_, the bacteria still grew slowly, albeit at a slower rate compared to the Blank + H_2_O_2_ group (Figure 4c–f). On the contrary, DT‐ZnFe‐LDH@Cu effectively inhibited the proliferation of bacteria, as evidenced by the flat curve over the full 24‐h period, highlighting the potential of DT‐ZnFe‐LDH@Cu to effectively prevent the formation and growth of biofilms.

The prevalence of bacterial infectious diseases associated with biofilms is significant in clinical settings. Due to the protective nature of EPS in the biofilm, treating infections associated with biofilms, especially those caused by drug‐resistant bacteria, is a complex task. Encouraged by the excellent bactericidal properties of DT‐ZnFe‐LDH@Cu, we conducted crystal violet staining assays to assess the ability of DT@Cu, DT‐ZnFe‐LDH, and DT‐ZnFe‐LDH@Cu to destroy bacterial biofilms. When DT@Cu and DT‐ZnFe‐LDH were exposed to H_2_O_2_, they showed limited biofilm clearance compared to the control (H_2_O_2_ alone) (Figure 4g,h). It was worth noting that DT‐ZnFe‐LDH@Cu + H_2_O_2_ condition removed more than 85% of the biofilms under the same conditions, indicating that the DT‐ZnFe‐LDH@Cu nanozyme effectively penetrated and eradicated the biofilm. These outcomes were in accordance with previous reports, suggesting DT‐ZnFe‐LDH@Cu effectively targeted and removed biofilms. The ability of the DT‐ZnFe‐LDH@Cu nanozyme to eradicate the biofilms was attributed to the Dex‐NH_2_ shells,^[^ ^33^ ^]^ which enabled the nanozyme to infiltrate deep biofilms and attach to the bacterial surface, causing the bacteria to detach from the biofilm. Once the nanozyme was embedded within the biofilm matrix, interactions between the nanozyme molecules and exogenous H_2_O_2_ generated ROS, thereby disrupting the integrity of the bacterial structure and ultimately killing the bacteria (Figure 4l).

To verify the penetration and accumulation of DT‐ZnFe‐LDH@Cu in mature biofilm, *P. aeruginosa* biofilm was employed as a model. After the biofilm was exposed to a Rhodamine B (RhoB)‐labeled DT‐ZnFe‐LDH@Cu suspension, confocal laser scanning microscopy (CLSM) was utilized to image *P. aeruginosa* within the biofilm through SYTO‐9 staining.^[^ ^39^, ^41^, ^42^, ^43^ ^]^ As shown in Figure 4k, the DT‐ZnFe‐LDH@Cu labeled with red fluorescence could effectively penetrate biological membranes, indicating that DT‐ZnFe‐LDH@Cu could diffusely spread throughout the entire biological membrane. This was attributed to the characteristic of the dextran domain in DT‐ZnFe‐LDH@Cu, which binded to the biological membrane matrix through bacterial extracellular enzymes. This observation was consistent with previous reports that dextran‐functionalized nanomaterial could target and penetrate biological membranels.^[^ ^33^, ^44^ ^]^

SEM was employed to examine changes in bacterial morphology and cell membrane integrity in the presence of various nanomaterials. When untreated, the bacterial membrane and cytoplasm remained intact (Figure 4i), while the SEM images of the bacteria showed that the cell walls and membranes of the bacteria treated with DT‐ZnFe‐LDH@Cu + H_2_O_2_ had fragmentated, causing the barriers to collapse and the cells to break apart and swell (Figure 4i, red arrow). Lastly, the ROS levels in *P. aeruginosa* exposed to the nanomaterials were measured using the 2′,7′‐dichlorodihydrodiacetate (DCFH‐DA) probe (Figure 4j). The bacterial cells treated with the control produced very few ROS, while the bacterial cells treated with DT@Cu and DT‐ZnFe‐LDH were stained green, indicating that the DCFH‐DA probe had reacted with ROS generated by the nanomaterials. The strongest green fluorescence was observed in the DT‐ZnFe‐LDH@Cu + H_2_O_2_ group, indicating that the total level of ROS in the bacteria was significantly higher than in the other groups. In addition, we quantitated the levels of ROS based on the fluorescence intensity of the DCFH‐DA probe in *P. aeruginosa*. The result indicated that following treatment with DT‐ZnFe‐LDH@Cu, the ROS levels in *P. aeruginosa* were the highest compared to the other materials (Figure S7, Supporting Information).

### 2.5 In Vitro Biosafety of DT‐ZnFe‐LDH@Cu

**Figure 5:**
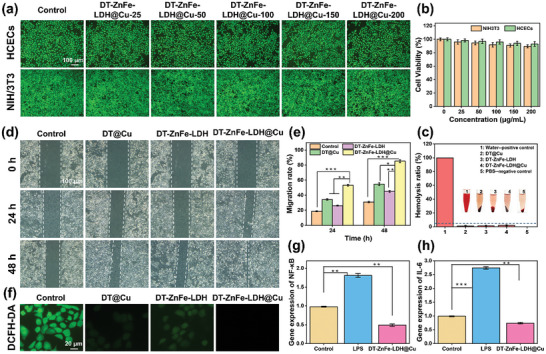
a,b) The FDA staining images and the cell viability of HCECs and NIH/3T3 and after culturing with DT‐ZnFe‐LDH@Cu (different concentrations of ZnFe‐LDH@Cu) for 24 h. c) Hemolysis assay of red blood cells treated with different treatments. d,e) The migration and the corresponding quantitative analysis results of HCECs cells in the culture plate after different treatments. f) Fluorescent images of LPS‐induced ROS production in RAW264.7 cells after different treatments. g,h) RT‐qPCR analysis of NF‐κB g) and IL‐6 h). Data are presented as mean ± SD (*n* = 3). (*p < 0.05, **p < 0.01, and ***p < 0.001).

The biocompatibility of DT‐ZnFe‐LDH@Cu was evaluated by fluorescein diacetate (FDA) staining of HCEC and NIH/3T3 cells. After incubation with different concentrations of DT‐ZnFe‐LDH@Cu (25, 50, 100, 150, and 200 µg mL^−1^) for 24 h, strong green fluorescence was observed in HCEC and NIH/3T3 cells (**Figure** **5**a), confirming that DT‐ZnFe‐LDH@Cu had no significant effect on the viability of HCEC and NIH/3T3 cells. The cell survival rate was still above 85%, even at the highest concentration of DT‐ZnFe‐LDH@Cu (200 µg mL^−1^) (Figure 5b). To demonstrate the biosafety (i.e., blood compatibility) of DT‐ZnFe‐LDH@Cu, we conducted in vitro hemolysis tests. As shown in Figure 5c, red blood cells from the whole blood of white rabbits were exposed to PBS (negative control), DT@Cu, DT‐ZnFe‐LDH, DT‐ZnFe‐LDH@Cu (100 µg mL^−1^), and water (positive control). Photographs of the whole blood following exposure to each group showed that DT@Cu, DT‐ZnFe‐LDH, DT‐ZnFe‐LDH@Cu, and PBS turned the blood light yellow, while the positive control was bright red, indicating serious hemolysis. When referenced to the positive control (100% hemolysis), the hemolysis of DT@Cu, DT‐ZnFe‐LDH, and DT‐ZnFe‐LDH@Cu did not exceed the 5% limit, indicating that the hemolysis was negligible for all three of these nanomaterials. This was further demonstrated by increasing the concentrations of DT‐ZnFe‐LDH@Cu beyond 200 µg mL^−1^. The whole blood was exposed to 500, 1, 1.5, and 2 mg mL^−1^ DT‐ZnFe‐LDH@Cu, and the hemolysis ratio of DT‐ZnFe‐LDH@Cu even at a concentration of up to 2 mg mL^−1^ was only 5.08% (Figure S8, Supporting Information). This finding indicated that even at elevated concentrations, DT‐ZnFe‐LDH@Cu exhibited good biosafety and did not appear to adversely affect biological systems.

### 2.6 DT‐ZnFe‐LDH@Cu Promotes Migration of HCEC cells

The scratch test was used to evaluate whether DT‐ZnFe‐LDH@Cu could improve the migration of HCECs within 48 h (Figure 5d). Compared to the control group (no material added), DT‐ZnFe‐LDH@Cu significantly promoted cells migration, with migration rates of 53.57% and 84.70% after 24 and 48 h, respectively, which was significantly higher than the control group (31.03%) (Figure 5e). The ability of DT‐ZnFe‐LDH@Cu to promote cells migration was mainly attributed to the presence of Cu^2+^—which can be utilized by Cu‐dependent enzymes or protein to play a signaling function to promote HCECs migration,^[^ ^45^, ^46^ ^]^ and Zn^2+^—which promotes enzyme catalysis and protein folding, and regulates gene expression.^[^ ^47^, ^48^ ^]^ The ability of the nanozyme to promote cell migration might also play a key role in corneal healing.

### 2.7 In Vitro Anti‐Inflammatory Effect of DT‐ZnFe‐LDH@Cu

The complex pathophysiology of the inflammatory response caused by BK had a significant influence on the postoperative microenvironment. Therefore, modulating the inflammatory response has become a key focus in developing ideal and effective drugs for the treatment of BK. Lipopolysaccharides (LPS) are a common endotoxin secreted by bacteria that induce oxidative stress in macrophages. To assess the anti‐inflammatory activity of DT‐ZnFe‐LDH@Cu, we measured the changes in ROS production in LPS‐induced macrophages (RAW264.7 cells) using the DCFH‐DA probe. As shown in Figure 5f and Figure S9 (Supporting Information), strong green fluorescence was detected from DCFH‐DA in the LPS‐stimulated macrophages in the control group, indicating the production of a large amount of ROS. After the addition of DT@Cu and DT‐ZnFe‐LDH, the cells emitted weak green fluorescence, indicating a decrease in ROS level. However, after treatment with DT‐ZnFe‐LDH@Cu, there was basically no fluorescence detected in the RAW264.7 cells. Furthermore, to evaluate the effect of DT‐ZnFe‐LDH@Cu on the immunoregulatory function of macrophages in vitro, we cultured RAW 264.7 cells with the DT‐ZnFe‐LDH@Cu. The M1 phenotype induced by LPS served as a positive control, while the untreated group served as a negative control. Real‐time quantitative polymerase chain reaction (qPCR) was utilized to quantify the expression levels of inflammatory factors (Table S6, Supporting Information). As illustrated in Figure 5g,h, the mRNA expression levels of inflammatory factors interleukin‐6 (IL‐6) and nuclear factor kappa B (NF‐κB) were significantly elevated in the LPS group compared to the control group. However, DT‐ZnFe‐LDH@Cu exhibited a notable attenuation of the expression of these inflammatory factors. We attributed the significant reduction in ROS production to the CAT‐like activity of DT‐ZnFe‐LDH@Cu, which could convert H_2_O_2_ into O_2_, thereby eliminating excess ROS and reducing oxidative stress.

### 2.8 In Vivo Therapeutic Effect of DT‐ZnFe‐LDH@Cu

**Figure 6:**
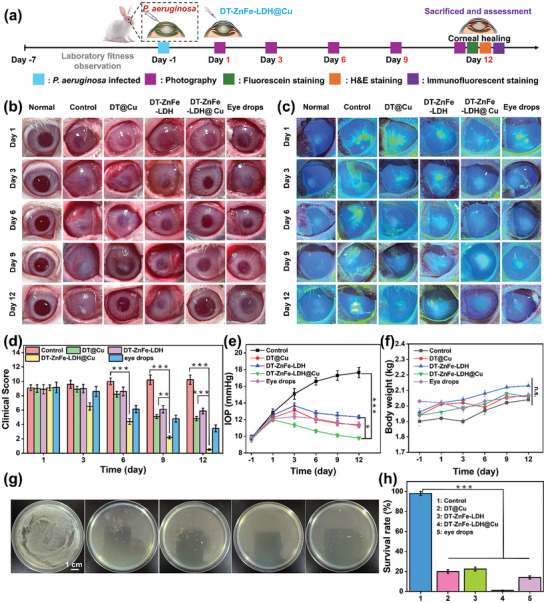
The therapeutic efficacy evaluation of the DT‐ZnFe‐LDH@Cu for bacterial keratitis. a) Schematic illustration of the establishment, administration, and sacrifice of the *P. aeruginosa*‐infected keratitis model. b) Representative slit‐lamp microscopy image appearance of *P. aeruginosa*‐infected keratitis. c) Fluorescein staining of the cornea under different administrations. d) Three volunteers assessed the rabbit eye surface with a corresponding clinical score (0‐12) based on three criteria (cloudy area, surface regularity, opacity density) and quantified the clinical score. e) IOP of rabbits after different treatments. f) Changes in the body weight of rabbits during different treatments. g) Photographs of bacterial colonies formed on TSB‐agar plates from corneal tissues. h) Quantitative analysis of bacterial survival rate on corneal surface. Data are presented as mean ± SD (*n* = 3). (n.s.: not significant, *p < 0.05, **p < 0.01, and ***p < 0.001).

A rabbit BK model was established by injecting 50 µL of *P. aeruginosa* (1 × 10^6^) into damaged cornea to evaluate the in vivo therapeutic effect of DT‐ZnFe‐LDH@Cu (**Figure** **6**a). Rabbits were divided into five groups (*n* = 3 in each group): control group, DT@Cu group (100 µg mL^−1^), DT‐ZnFe‐LDH group (100 µg mL^−1^), DT‐ZnFe‐LDH@Cu group (100 µg mL^−1^), and tobramycin antibiotic eye drops group. Treatments were administered three times a day for the first 5 days and twice a day from day 6 to 10, with photographs of the eye taken every 3 days. A clinical slit‐lamp was used to monitor changes to the corneal morphology, abscess, and inflammation over the 12‐day treatment window (Figure 6b,d). Scores were evaluated based on edema, surface ulceration, opaque area; a low score generally indicates high efficacy of the nanomaterial for treating BK. One day after infection with *P. aeruginosa*, corneal edema, ulceration, and dense turbidity appeared in all rabbits, almost completely covering the cornea. Clinical total scores were high, indicating the successful development of BK.

During the course of treatment, it was observed that the rabbits in the control group suffered from severe infection, marked corneal opacity, and significant inflammation persisting until day 12, with the highest clinical score recorded (10.9 ± 0.4). While the development of the corneal infection was partially controlled in the DT@Cu and DT‐ZnFe‐LDH groups, clarity of the cornea did not fully return, and inflammation persisted on day 12 (DT@Cu = 4.8 ± 0.10, DT‐ZnFe‐LDH = 5.9 ± 0.12). Although there were notable improvements in the abscesses and transparency of the corneal ulcer in the group treated with eye drops alone, severe inflammation and edema still persisted after 12 days of treatment, with an inflammation score remaining at 3.9 ± 0.2. The cornea in the DT‐ZnFe‐LDH@Cu group gradually cleared on day 3, achieving substantial clarity by day 6 without any abscess formation. Complete wound recovery occurred by day 9, and by day 12, high transparency and subsided inflammation were observed, signifying a phenotype consistent with that of normal eyes. Furthermore, on day 12, the rabbits in the DT‐ZnFe‐LDH@Cu group demonstrated the lowest eye clinical score (clinical score = 0.5 ± 0.25). Corneal integrity was evaluated using fluorescein sodium staining. As depicted in Figure 6c, after 12 days post‐treatment, the control group exhibited substantial green fluorescence, indicative of severe infiltration of inflammatory cells and significant corneal defect. Conversely, in the DT@Cu and DT‐ZnFe‐LDH groups, the green fluorescence diminished and the defect area decreased. Notably, fluorescence in the DT‐ZnFe‐LDH@Cu treatment group was minimal, suggesting intact cornea devoid of inflammation, indicating that DT‐ZnFe‐LDH@Cu effectively inhibited corneal opacity progression and defect severity.

Intraocular pressure (IOP) was monitored every 3 days during treatment (Figure 6e). The IOP of the control group increased gradually, and the IOP value was 17.5 ± 0.36 mmHg on day 12. The IOP value of the DT@Cu and DT‐ZnFe‐LDH treatment groups increased slightly from 12.11 to 13.14 mmHg and 12.54 to 13.63 mmHg between days 1 and 3 and then gradually decreased and recovered to 11.64 and 12.52 mmHg by day 9, reaching 11.5 ± 0.15 mmHg and 12.46 ± 0.17 mmHg on day 12, respectively. The IOP value for the DT‐ZnFe‐LDH@Cu group on day 12 stayed consistent at 9.82 ± 0.1 mmHg, highlighting the superiority of the DT‐ZnFe‐LDH@Cu in reducing IOP. At the conclusion of the treatment, bacteria were harvested from the ocular surface and quantified by plate counting. The control group exhibited the highest number of bacterial colonies, whereas the DT@Cu and DT‐ZnFe‐LDH demonstrated partial eradication of bacterial colonies. Antibiotic eye drops also had a noticeable antibacterial effect, while DT‐ZnFe‐LDH@Cu nearly eliminated all bacterial colonies (Figure 6g). As depicted in Figure 6h, the bacterial survival rates were 97.4% in the control group, 20.0% in the DT@Cu group, 22.5% in the DT‐ZnFe‐LDH group, 14.3% in the antibiotic eye drops group, and 1.2% in the DT‐ZnFe‐LDH@Cu group. Furthermore, there was no significant variance observed in body weight across groups (Figure 6f). These results confirmed that DT‐ZnFe‐LDH@Cu could effectively treat *P. aeruginosa* infection in the cornea, providing a new strategy for the clinical treatment of BK.

**Figure 7:**
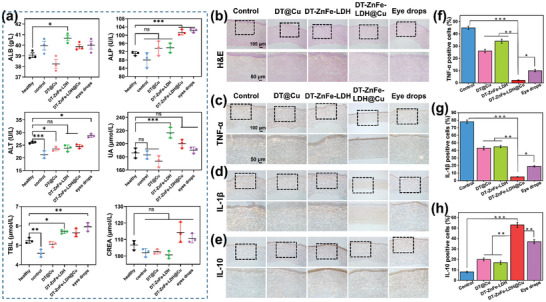
a) Blood biochemical indexes of liver function and kidney function. ALT: alanine aminotransferase, ALP: alkaline phosphatase, ALB: albumin, TBIL: total bilirubin, UA: uric acid, and CREA: creatinine. b) H&E staining images of cornea in different treatment groups. Immunohistochemical images of TNF‐α c), IL‐1β d), and IL‐10 e). Quantitative analysis of immunohistochemical positive cells after different treatments: TNF‐α f), IL‐1β g), and IL‐10 h). Data are presented as mean ± SD (*n* = 3). (ns: not significant, *p < 0.05, **p < 0.01, and ***p < 0.001).

To assess the in vivo toxicity of DT‐ZnFe‐LDH@Cu, we injected rabbits with DT‐ZnFe‐LDH@Cu (100 mg mL^−1^) into the cornea for 12 days, after which whole blood was collected from the rabbit following euthanasia, and blood biochemical analysis was performed. To assess liver function, we quantified the levels of alanine aminotransferase (ALT), alkaline phosphatase (ALP), albumin (ALB), and total bilirubin (TBIL), and to assess renal function, we quantified the levels of uric acid (UA) and creatinine (CREA). As presented in **Figure** **7**a, there were no significant abnormal changes in liver function and renal function indices between the control, DT@Cu, DT‐ZnFe‐LDH, DT‐ZnFe‐LDH@Cu, and eye drops treatment groups and the healthy group (control), indicating that all nanomaterials had minimal‐to‐no effect on the physiological function of the liver or kidneys.

### 2.9 Histological Analysis

Histological analysis of the rabbit cornea was conducted using H&E staining to further assess the effects of the different nanomaterials on corneal healing. In the control group, significant inflammatory cell infiltration was observed (Figure 7b). However, in the rabbits that were treated with DT@Cu, DT‐ZnFe‐LDH, and eye drops, inflammatory cell infiltration had been reduced, as demonstrated by bright components of tissue. Notably, inflammation in the corneas of the DT‐ZnFe‐LDH@Cu group was largely suppressed, the epidermis remained intact, and corneal collagen fibroadenoma had been significantly reduced (Figure 7b). To further validate the outcomes of the histological analysis, immunohistochemical staining was conducted in each group by measuring the changes in the expression levels of TNF‐α, IL‐1β, and IL‐10, all of which are associated with inflammation. As depicted in Figure 7c–h, the expression levels of the pro‐inflammatory factors TNF‐α and IL‐1β in the DT‐ZnFe‐LDH@Cu group were the lowest compared to the control group, while the expression levels of the anti‐inflammatory factor IL‐10 was the highest, indicating that DT‐ZnFe‐LDH@Cu most effectively suppressed inflammation, enabling the nanomaterial to mitigate infection caused by bacteria.

**Figure 8:**
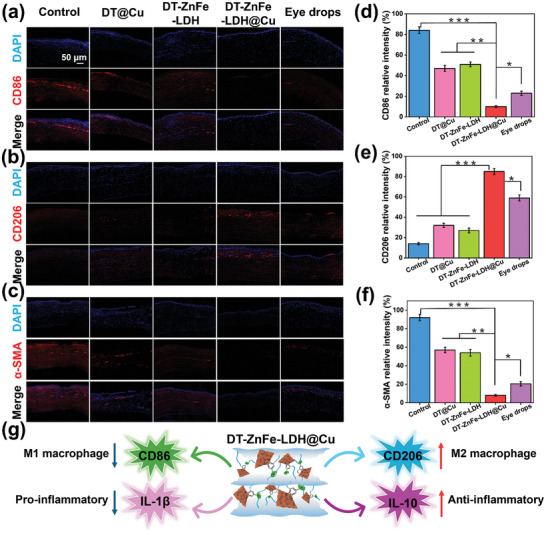
Immunofluorescence images of CD86 a), CD206 b), and α‐SMA c) in different treatment groups. Cell nuclei were stained with DAPI (blue). Relative statistical analysis of CD86 d), CD206 e), and α‐SMA f) with different treatment groups. g) Schematic diagram of the mechanism of DT‐ZnFe‐LDH@Cu mediating the polarization of macrophages from M1 type to M2 type, with the decrease of pro‐inflammatory factors and the increase of anti‐inflammatory factors. Data are presented as mean ± SD (*n* = 3). (*p < 0.05, **p < 0.01, and ***p < 0.001.).

Macrophages play a central role in the regulation of inflammation and can be divided into classical pro‐inflammatory macrophages (M1) and anti‐inflammatory macrophages (M2). CD86 and CD206 are typical biomarkers of M1 and M2 macrophages, respectively, and their levels in infected tissues reflect the inflammatory status. To validate the results of the previous histological analysis, we sought to assess the changes in levels of the inflammatory biomarkers CD86 and CD206 in infected corneal tissues by immunofluorescence staining. As shown in **Figure** **8**a,d, red fluorescence was observed in the control, DT@Cu, DT‐ZnFe‐LDH, and eye drop groups, indicating that CD86 proteins were overexpressed. When overlayed with the signal from DAPI, a fluorescent dye that stains DNA in the nuclei, the red fluorescence signal was still clearly observed. However, the expression of CD86 was significantly downregulated in the DT‐ZnFe‐LDH@Cu treatment group. Subsequent anti‐inflammatory marker staining analysis revealed that there was nearly no expression of CD206 (red fluorescence) in the control, DT@Cu, and DT‐ZnFe‐LDH treatment groups. Especially when compared to eye drops, the DT‐ZnFe‐LDH@Cu group significantly upregulated the expression of CD206 (red fluorescence) in the infected tissues (Figure 8b,e), implying that macrophages gradually polarized from the M1 phenotype to the M2 phenotype during recovery from infected cornea treatment, further demonstrating that DT‐ZnFe‐LDH@Cu was an effective inhibitor of inflammation in the treatment of BK (Figure 8g).

During wound healing, scar tissue formation can negatively impact corneal remodeling and ultimately impair vision. Hence, we evaluated the ability of DT‐ZnFe‐LDH@Cu to regulate corneal wound hyperplasia by measuring the changes in the expression of α‐SMA, a distinctive marker of corneal myoblasts (MFB),^[^ ^49^ ^]^ by immunofluorescence staining. As shown in the fluorescence images and corresponding quantitative data in Figure 8c,f, respectively, α‐SMA (red fluorescence) was not significantly expressed in the corneal tissue of the rabbits treated with DT‐ZnFe‐LDH@Cu, indicating that myofibroblast activation was not elicited. In contrast, in the control group, α‐SMA expression was high, suggesting that myofibroblasts were highly activated, which might be associated with the persistence of inflammation, thereby eliciting a considerable immune response, that observed in the control group. The reduction in inflammation during the corneal healing process enabled restoration of the cornea to normal function and morphology without the formation of a scar, corroborating the potential of DT‐ZnFe‐LDH@Cu as an effective treatment for BK.

## 3 Conclusion

The results reported herein highlight the novelty and capability of the DT‐ZnFe‐LDH@Cu nanozyme as an antibacterial and anti‐inflammatory therapy for the treatment of BK. The unique triple enzyme‐like catalytic activities of the DT‐ZnFe‐LDH@Cu was enabled by the loading of Cu‐SAzymes and Dex‐NH_2_ onto ZnFe‐LDH. Furthermore, the Dex‐NH_2_ on the surface enabled the DT‐ZnFe‐LDH@Cu to penetrate bacterial biofilms and adhered the biofilm via electrostatic interactions. Under acidic conditions, the POD‐like and OXD‐like activities of DT‐ZnFe‐LDH@Cu catalyzed the conversion of H_2_O_2_ to ^•^OH, O_2_^•−^, and ^1^O_2_, thereby killing the bacteria. Simultaneously, the CAT‐like activity enabled the nanozyme to convert excess ROS into O_2_, thereby alleviating hypoxia and regulating inflammatory responses. Notably, DT‐ZnFe‐LDH@Cu demonstrated superior therapeutic potential in treating bacterial infections in the corneas of a BK rabbit model compared to commercially available tobramycin eye drops. This was mediated, at least in part, by the downregulation of pro‐inflammatory factors TNF‐α and IL‐1β and the upregulation of the anti‐inflammatory factor IL‐10, as well as downregulation of the expression of CD86 and upregulation of the expression of CD206, enabling polarization of macrophages from M1 to M2 to further reduce inflammation. DT‐ZnFe‐LDH@Cu also downregulated the expression of α‐SMA, thereby preventing scar formation and facilitating corneal healing. Additionally, to ensure stability of the material, DT‐ZnFe‐LDH@Cu should be stored in a sealed container at low‐temperature in the dark to prevent exposure to high humidity and significant environmental fluctuations. Moreover, the synthetic method developed in this study was straightforward, and the raw materials were inexpensive and readily accessible, making it feasible for practical industrial production applications in the future. All in all, DT‐ZnFe‐LDH@Cu demonstrated significant potential as a clinically relevant therapy for the treatment of BK.

## 4 Experimental Section

### Synthesis of ZnFe‐LDH

ZnFe‐LDH was synthesized using a straightforward co‐precipitation method. The metal salt mixture consisted of Fe(NO_3_)_3_·9H_2_O (1 mmol L^−1^) and Zn(NO_3_)_2_·6H_2_O (2 mmol L^−1^). And NaOH (0.35 mol L^−1^) and Na_2_CO_3_ (0.15 mol L^−1^) were prepared by dissolving the salts in 100 mL of water. Under continuous magnetic stirring, this solution was gradually dropped into the metal salt mixture, maintaining the pH of the suspension at 10 ± 0.05. Once precipitation was formed, the suspension was stirred for an additional 30 min. The suspension underwent hydrothermal aging at 65 °C for 24 h. After cooling to room temperature, the final product was washed with ultrapure water five times and then frozen‐dried for 24 h to obtain the powder of ZnFe‐LDH.

### Synthesis of ZnFe‐LDH@Cu

The precursor of Cu‐SAzymes was synthesized according to previous reports.^[^ ^36^ ^]^ The nitrogen‐free Cu‐BTC MOF (Cu(BTC)(H_2_O)_3_) precursor was synthesized in an aqueous solution via a straightforward chemical precipitation method using copper acetate and 1,3,5‐benzoic acid as raw materials. Specifically, Cu(CH_3_COO)_2_·H_2_O (0.398 g) was dissolved in 100 mL aqueous solution. In a separate container, 1,3,5‐benzoic acid (0.232 g) was dissolved in another 100 mL aqueous solution. These two solutions were subsequently combined and stirred at room temperature for 2 h. The resulting solid product was then collected through centrifugation, thoroughly cleaned with water, and then freeze‐dried for 24 h. This solid was further ground together with dicyandiamide until a uniform mixture was obtained.

Subsequently, the ZnFe‐LDH powder was evenly mixed with the solution of the above mixture. The resulting mixture was subjected to ultrasound treatment in an ice water bath for 15 min, after which it was immediately frozen in the refrigerator and freeze‐dried in the freeze‐dryer. The resulting powder was ground and transferred into a corundum boat, which was then placed in a vacuum tube furnace. The material was heated under H_2_/Ar protection at a rate of 80 °C min^−1^ to 600 °C for sintering for 2 h. The temperature was then decreased at a rate of 30 °C per min, during carbonization, pyrolysis, and nitriding processes occurred. After heat treatment, the pyrolysis products were washed with a substantial amount of deionized water and dilute acid under ultrasound to remove dicyandiamide and metal residues, and the final powder was ZnFe‐LDH@Cu.

### Synthesis of DT‐ZnFe‐LDH@Cu

Dex‐NH_2_ was synthesized according to the method reported by previous researchers.^[^ ^33^, ^50^ ^]^ Using *N*, *N*′‐carbonyl diimidazole (CDI) as an activator, a cationic dextran was prepared by coupling ethylenediamine with the hydroxyl group of dextran. Simply, dextran (0.5 g) was dissolved in dimethyl sulfoxide (DMSO) (25 mL) and incubated with CDI (1.0 g) under N_2_ conditions. The degree of cationization was directly proportional to the mass ratio of CDI to dextran. After 2 h, ethylenediamine (2.5 mL) was added to facilitate crosslinking. The mixture was allowed to react at room temperature under nitrogen for 24 h, followed by dialysis with deionized water for 3 days. After drying, Dex‐NH_2_ product would be obtained.

Subsequently, ZnFe‐LDH@Cu was treated ultrasonically in 5 mL of deionized water (150 W, 20 kHz) for 10 min. The TA was dissolved in pure water, and the TA solution was mixed with ZnFe‐LDH@Cu suspension, which was then subjected to ultrasonic oscillation for 30 min followed by stirring at room temperature for 2 h to achieve a uniform suspension. Finally, the previously obtained Dex‐NH_2_ product was added to this suspension to obtain DT‐ZnFe‐LDH@Cu.

### Peroxidase‐Like Properties of ZnFe‐LDH@Cu

To investigate the POD‐like activity of ZnFe‐LDH@Cu, TMB was employed as an indicator for the detection of ^•^OH. The experimental conditions were as follows: samples of ZnFe‐LDH, Cu‐SAzymes, ZnFe‐LDH@Cu, and DT‐ZnFe‐LDH@Cu (100 µg mL^−1^) were mixed with TMB in 1 mL of PBS (pH 5.5). Subsequently, H_2_O_2_ (1 mm) was added to the solution. The absorbance of oxTMB at 652 nm was measured by UV–vis spectroscopy at regular intervals.

To further assess the activity kinetics of POD‐like enzymes, varying concentrations of substrate (TMB (0.1–1.0 mm) or H_2_O_2_ (10–60 mm)) were introduced while maintaining the concentration of ZnFe‐LDH@Cu constant at 100 µg mL^−1^. The steady‐state reaction rate at these different substrate concentrations was determined by analyzing the slope of the initial absorbance over time.

The absorbance value at 652 nm was converted to the concentration of oxTMB according to Lambert–Beer law.

(1) $$V=\frac{k}{\varepsilon \times b}$$

where *V* is the initial velocity, ε = 3.9 × 10^4^ M^−1^ cm^−1^, b = 1 cm, *k* = ΔA/Δt.

The Michaelis–Menten curve was drawn by calculating the relationship between *V* and substrate concentration. The apparent kinetic parameters *K_m_* and *V_max_* were calculated according to the Michaelis–Menten curve with the following formula:

(2) $$v_{0}=\frac{V_{max}\times \left[S\right]}{K_{m}+\left[S\right]}$$

where *V* is the initial velocity, *V_max_* is the maximal reaction velocity, [*S*] is the concentration of substrate and *K_m_* is the Michaelis constant, which represents the concentration of the substrate when the *V* reaches half of the *V_max_*.

### Catalase‐Like Properties of ZnFe‐LDH@Cu

The oxygen production of ZnFe‐LDH@Cu was assessed using a portable dissolved oxygen meter (JPBJ‐608) to assess its CAT‐like activity. For this evaluation, ZnFe‐LDH, Cu‐SAzymes, and ZnFe‐LDH@Cu (all at a concentration of 100 µg mL^−1^) were combined with H_2_O_2_ (1 mm) in a PBS solution. The oxygen concentration was recorded in real time over 10 min.

To conduct the kinetic analysis of ZnFe‐LDH@Cu using H_2_O_2_ as substrate, different concentrations of H_2_O_2_ (10–60 mm) solution were sequentially added. The changes in oxygen concentration corresponding to each H_2_O_2_ concentration were used to determine the initial reaction rate (*V_0_*). The reaction rate data were then fitted to the Michaelis–Menten curve (Equation 2) to derive the *K_m_* and *V_max_*.

### Oxidase‐Like Properties of ZnFe‐LDH@Cu

OXD‐like kinetic analysis of ZnFe‐LDH@Cu with TMB as the substrate. Different concentrations of TMB (0.1, 0.2, 0.4, 0.6, 0.8, and 1 mm solution was added to the reaction mixture, and the initial reaction rate (*V_0_*) was determined by measuring the change in absorbance under the Beer–Lambert Law (Equation 3). The reaction rates were plotted against TMB content and then fitted with the Michaelis–Menten curve (Equation 2), enabling the determination of the *K_m_* and *V_max_*.

(3) A=εbc

### Antibacterial Assay of DT‐ZnFe‐LDH@Cu In Vitro—Antibacterial Plate Counting Experiment In Vitro

To assess the broad‐spectrum antibacterial efficacy of DT‐ZnFe‐LDH@Cu (100 µg mL^−1^), *P. aeruginosa*, MRSA, *S. aureus*, and *E. coli* (1 × 10^6^ CFU) were incubated in Luria–Bertani (LB) medium at 37 °C for 24 h, respectively, to reach the logarithmic growth phase. Following this, the bacterial suspension was combined with PBS + H_2_O_2_ (0.1 mm), DT@Cu + H_2_O_2_ (0.1 mm), DT‐ZnFe‐LDH + H_2_O_2_ (0.1 mm), and DT‐ZnFe‐LDH@Cu + H_2_O_2_ (0.1 mm) groups, and incubated at 37 °C for an additional 24 h. The bacterial solution was then diluted 10 000 times, and 50 µL of this diluted bacterial solution was plated onto solid medium. The plates were incubated at 37 °C for 24 h to monitor bacterial growth, with each condition replicated three times. The CFU numbers of *P. aeruginosa*, MRSA, *S. aureus*, and *E. coli* were counted respectively.

### Antibacterial Assay of DT‐ZnFe‐LDH@Cu In Vitro—Assessment of Biofilm Resistance Using Crystal Violet Staining

To investigate biofilm inhibition, *P. aeruginosa*, MRSA, *S. aureus*, and *E. coli* (1×10^6^ CFU) were diluted with liquid medium to OD_600_ = 0.05. The suspension was transferred to wells of the 12‐well plate and cultured in an incubator at 37 °C for 24 h to allow biofilm formation. Following the establishment of the bacterial biofilm, the cultures were subjected to the same as described in the in vitro antibacterial experiment. Incubation continued for another 24 h at 37 °C to facilitate further bacterial biofilm growth. Afterward, the medium and suspended bacteria were carefully removed, and the wells were gently rinsed with PBS without damaging the established biofilm. The bacterial biofilm was fixed with 95% formalin for 15 min, stained with 0.1% crystal violet solution for 20 min, and then washed gently with PBS for 3 times to remove excess dye. The wells were then air‐dried for 1 h. Finally, 200 µL of 33% glacial acetic acid were added to evaluate the crystal violet stained biofilm.

### Antibacterial Assay of DT‐ZnFe‐LDH@Cu In Vitro—Biofilm Penetrated and Elimination Assay

RhoB was employed to label DT‐ZnFe‐LDH@Cu (100 µg mL^−1^) materials. To put it simply, *P. aeruginosa* (1 × 10^6^ CFU) suspension was added to 2 mL Tryptone Soya Broth (TSB) medium and cultivated at 37 °C for 3 days to form a biofilm. Subsequently, the suspension was aspirated from the petri dish and washed twice with PBS. Then, PBS and DT‐ZnFe‐LDH@Cu were added respectively to the Petri dishes. After incubation at 37 °C for 2 h, the biofilm was gently washed with PBS twice to eliminate non‐adhering substances. Prepared the staining working solution (with SYTO‐9 at a working concentration of 5 µm), and added 500 µL of the staining working solution dropwise to the surface of the biological membrane, followed by incubation in the dark at 37 °C for 15 min. After staining, washed the surface of the sample with sterile PBS solution and captured images using a CLSM at 200 × magnification.

### In Vitro Cell Migration of DT‐ZnFe‐LDH@Cu

HCECs were inoculated in a 24‐well plate (1 × 10^4^ cells per well) and cultured at 37 °C and 5% CO_2_ for 24 h. A straight line was scratched across each well using a 200 µL pipette tip, and the wells were subsequently washed with PBS to remove any debris. The cells were incubated in the untreated, DT@Cu, DT‐ZnFe‐LDH, and DT‐ZnFe‐LDH@Cu (100 µg mL^−1^) experimental groups in the medium at 37 °C and 5% CO_2_ for 24 h. Photographs of each well were taken to calculate cell mobility. The cells were passaged every 3 days using 0.25% trypsin containing 0.02% EDTA.

### In Vitro Anti‐Inflammatory Effect of DT‐ZnFe‐LDH@Cu

1. The DCFH‐DA probe was used to detect the production of ROS in cells. In short, RAW264.7 cells were seeded in a six‐well plate at a density of 1 × 10^4^ cells per well and incubated in a humidified atmosphere containing 5% CO_2_ for 24 h. Following this incubation, the medium was removed, and the cells were washed twice with PBS. The cells were then treated with different components, with H_2_O_2_ added to induce ROS production. After the above treatment, DCFH‐DA was diluted in serum‐free medium at a ratio of 1:1000 to achieve a final concentration of 20 µm. The cell culture medium was removed, and an adequate volume of the diluted DCFH‐DA were added to ensure complete coverage of the cells, Typically, 1 mL of diluted DCFH‐DA was used for each well of the six‐well plate. The cells were incubated at 37 °C for 20 min, after which the medium was removed, and the cells were washed three times with PBS. Fluorescence was then observed using fluorescence microscope, with an excitation wavelength of 488 nm and an emission wavelength of 525 nm.
2. RAW 264.7 macrophages were cultured in 12‐well plates and subjected to one of three treatments: 1) PBS, 2) LPS, and 3) DT‐ZnFe‐LDH@Cu (100 µg mL^−1^). Following a 24‐h co‐culture period, macrophages from each treatment group were lysed using Trizol, and total RNA was subsequently extracted. Complementary DNA (cDNA) was then synthesized using a cDNA reverse transcription kit. The expression levels of IL‐6 and NF‐κB were quantified using real‐time quantitative polymerase chain reaction (RT‐qPCR) with SYBR Green and appropriate primers (Table S5, Supporting Information).

### In Vivo Treatment of BK Infected with *P. aeruginosa*

The animal experiment program should be conducted in strict compliance with the requirements established by the Chinese National Standard GB/T35892‐2018 and the Animal Protection and Ethics Committee of Northwest A&F University (NWAFU. No20234580d0600601 [2000]). Animal handling protocols will adhere to the guidelines provided by the Association for Research in Vision and Ophthalmology (ARVO) regarding the use of animals in ophthalmic and vision research. New Zealand white rabbits (aged 4–6 weeks) were selected for this study, while those with pre‐existing ocular surface diseases were excluded. The rabbits were maintained in an alternating light and dark cycle in an environment at temperature of 25 ± 2 °C, appropriate humidity, and were provided with adequate drinking water and food on a 12/12 h schedule. After 1 week of adaptation, rabbits with BK could be established according to the standard protocol.

### In Vivo Treatment of BK Infected with *P. aeruginosa*— Establishment of BK Model in Rabbit

Initially, pentobarbital (50 mg kg^−1^) and hydrochloric acid eye drops (0.4%) were administered intraperitoneally. A scalpel was used to mark and scrape the interstitial layer of the cornea surface. Immediately afterward, 1 × 10^6^ of *P. aeruginosa* was evenly applied to the corneal defect using a sterile cotton swab. Following successful modeling, the white rabbits were randomly divided into five groups with three rabbits in each group: 1) PBS, 2) DT@Cu (100 µg mL^−1^), 3) DT‐ZnFe‐LDH (100 µg mL^−1^), 4) DT‐ZnFe‐LDH@Cu (100 µg mL^−1^), and 5) eye drops. In the control group, aseptic PBS was added to the corneal wounds of white rabbits on days 1–3 and 5–6 (1 drop in the morning and 1 drop in the evening each day) as well as on days 8–9 (1 drop at noon each day). The treatment group was injected DT@Cu, DT‐ZnFe‐LDH, DT‐ZnFe‐LDH@Cu, and eye drops on the corneal wounds of white rabbits on days 1–3, 5–6 (1 drop in the morning and evening each day) and on days 8–9 (1 drop at noon each day), respectively. Corneal infection and pathological features were observed under slit‐lamp microscope and photographed on days 0, 1, 3, 5, 7, and 9. The severity of BK was assessed based on corneal opacity, density, and surface irregularity. Additionally, the body weight of rabbits in each group was measured on days 1, 3, 5, 7, and 9 of administration. Fluorescein staining was employed to assess the area of the epithelial defect.

### In Vivo Treatment of BK Infected with *P. aeruginosa*—Assessment of Corneal Surface Bacteria

In vivo antibacterial experiment: On the 10th day, the eyes of rabbits from each group were sacrificed, and their eyes were harvested. The corneas were carefully separated from the eyeballs on the super‐clean experimental table. Following this, the corneas were cut into small pieces and placed in 5 mL sterile PBS. From each sample, 50 µL PBS supernatant was spread on agar plate for bacterial culture. After 24 h of incubation, the number of bacteria was quantified by counting the individual colonies on the agar plate.

### In Vivo Treatment of BK Infected with *P. aeruginosa*—Histology, Immunohistochemistry, and Immunofluorescence Staining Evaluation

On the last day of treatment, corneal samples were collected. Then, H&E staining was performed to evaluate corneal edema, endoplasmic reticulum thickness, and inflammation. On the other hand, immunohistochemical staining was performed with TNF‐α, IL‐1β, and IL‐10, respectively, to evaluate the quantity of inflammatory cells. The contents of M1 macrophage marker CD86 and M2 macrophage marker CD206 were quantitatively analyzed through immunofluorescence staining to evaluate the polarization of M1 macrophages to M2 macrophages. Furthermore, using immunofluorescence staining with α‐SMA antibodies was used to evaluate whether there was neovascularization in the cornea.

### Statistical Analysis

The experimental data in this study were statistically analyzed, and the relevant results were reported as mean ± standard deviation (*n* = 3), in the case of statistically significant differences, and the statistical analysis was implemented using the GraphPad Prism 9 software. Unlike comparing multiple groups by one‐way variance analysis, the statistically significant differences between the two groups were compared by unpaired *t*‐tests. *p*‐values were calculated using ANOVA, and the levels of significance were labeled as non‐significant (n.s.), *(*p* < 0.05), **(*p* < 0.01), or ***(*p* < 0.001).

## Conflict of Interest

The authors declare no conflict of interest.

## Supporting information



Supporting Information

## Data Availability

The data that support the findings of this study are available from the corresponding author upon reasonable request.
