# Correction to “ZnFe Layered Double Hydroxide Nanosheets Loaded with Cu Single‐Atom Nanozymes with Multi‐Enzyme‐Like Catalytic Activities as an Effective Treatment for Bacterial Keratitis”

**DOI:** 10.1002/advs.202513019

**Published:** 2025-09-08

**Authors:** Keke Wang, Mao‐sen Yuan, Pengxiu Dai, Jing Li, Anju Tao, Xinke Zhang, Jinyi Wang, Qin Tu

https://doi.org/10.1002/advs.202411999

In Figure 5d, the images representing the Control group at 0 h and the DT‐ZnFe‐LDH group at 0 h are duplicates. The revised Figure 5 has been provided. This error occurred due to the excessive number of experimental images, which led to mistakes during the assembly of Figure 5. Importantly, this error does not impact the scientific conclusions of the study.


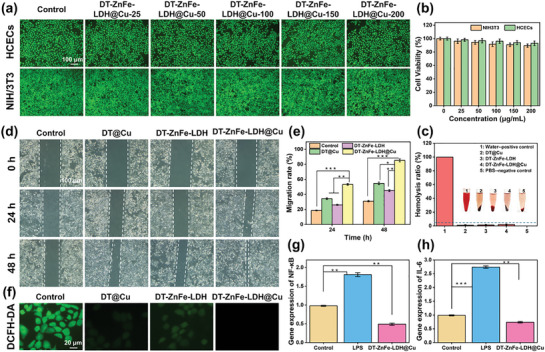


We apologize for this error.

