# Supplementary material for: ZnFe Layered Double Hydroxide Nanosheets Loaded with Cu Single‐Atom Nanozymes with Multi‐Enzyme‐Like Catalytic Activities as an Effective Treatment for Bacterial Keratitis
Source: Adv Sci (Weinh). 2025 Jan 22;12(10):2411999. doi: 10.1002/advs.202411999 (PMC11905092; doi:10.1002/advs.202411999)
Supplement: Supplementary file 1 — Supporting Information [file ADVS-12-2411999-s001.docx]

Supporting Information

**ZnFe Layered Double Hydroxide Nanosheets Loaded with Cu Single-Atom Nanozymes with Multi-enzyme-like Catalytic Activities as An Effective Treatment for Bacterial Keratitis**

Keke Wang^a,1^, Mao-sen Yuan^a,1^, Pengxiu Dai^b,1^, Jing Li^a^, Anju Tao^a^, Xinke Zhang^b,^*, Jinyi Wang^a,^*, and Qin Tu^a,^*

^a^ College of Chemistry and Pharmacy, Northwest A&F University, Yangling, Shaanxi 712100, P. R. China

^b^ College of Veterinary Medicine, Northwest A&F University, Yangling, Shaanxi 712100, P. R. China

*Corresponding author. E-mail address: tuqin@nwsuaf.edu.cn (Q. Tu)

**Experimental section**

*Materials*

Tannic acid, Cupric acetate monohydrate (Cu (CH_3_COO)_2_·H_2_O, purities ≥ 99.95 %), 1,3,5-benzoic acid and dicyandiamine were supplied by Titan Technology Co., Ltd., Shanghai, China. *N*, *N* '-carbonyl diimidazole, ferric nitrate ninahydrate (Fe(NO_3_)_3_·9H_2_O, 99 %) and zinc nitrate hexahydrate (Zn(NO_3_)_2_·6H_2_O, 99 %) were procured from Aladdin Biochemical Technology Co., Ltd., Shanghai, China. 3, 3′, 5, 5′-tetramethylbenzidine dihydrochloride hydrate (TMB, purities ≥ 98 %), rhodamine B (RhoB) isothiocyanate, 1,3-diphenylisobenzofuran (DPBF, purities ≥ 97 %), and 9, 10-anthracenedi-bis (methylene) dicarboxylic acid (ABDA, purities ≥ 90 %) were sourced from Maclin Biochemical Technology Co., Ltd., Shanghai, China. Ethylenediamine was obtained from Tianli Chemical Reagent Co., Ltd., Tianjin, China. 2′, 7′- dichlorofluorescindiacetate (DCFH-DA, purities ≥ 99 % or higher) was purchased from MedChemexpress biotechnology company, Shanghai, China, and dextran was provided by Anergy Chemical, Shanghai, China. SYTO-9 was purchased from Thermo Fisher. All these chemicals were of reagent grade or better, and used without further purification. This study utilized Gram-positive *Staphylococcus aureus* (*S. aureus*), Gram-negative *Escherichia coli* (*E. coli*), methicillin-resistant *Staphylococcus aureus* (MRSA), and *Pseudomonas aeruginosa* (*P. aeruginosa*). Human corneal epithelial cells (HCECs) were obtained from the Shaanxi Stem Cell Engineering Technology Research Center.

*Characterization*

Ultrahigh resolution field emission transmission electron microscopy (URFE-TEM, FEI Talos F200S, USA) confirmed the existence of single copper atoms. The morphologies of ZnFe-LDH@Cu nanosheets were characterized by transmission electron microscopy (TEM, HT7800, Japan). The particle size and zeta potential were measured at room temperature using a nano-laser particle size analyzer (ZEN3600, UK). X-ray diffractometry (XRD, D8 ADVANCE A25, Germany) was employed to analyze XRD patterns in the range of 2*θ* =10°-80°. The thickness of the ZnFe-LDH@Cu nanosheets was observed using Atomic Force microscope (AFM, Multimode-8, USA). The proportions of the metal components Zn, Fe, and Cu were determined by inductively coupled plasma atomic emission spectrometry (ICP-OES, ARCOS, Germany). The Brunauer-Emmett and Teller (BET) surface areas of ZnFe-LDH@Cu were investigated by nitrogen adsorption at 77 K (Micromeritics ASAP 2460, USA). The microscopic morphology of DT-ZnFe-LDH@Cu materials was observed by scanning electron microscope (SEM, ZEISS Sigma 300, Germany). The location and distribution of C, N, O, Zn, Fe and Cu in DT-ZnFe-LDH@Cu were determined by energy dispersive spectroscopy (EDS) coupled with SEM. Fourier transform infrared spectroscopy (FT-IR, Vertex70, Germany) was used to analyze the composition and identify the structure. X-ray photoelectron spectroscopy (XPS, Kratos, AXISSupra, UK) was applied to analyze the chemical composition of DT-ZnFe-LDH@Cu. The absorption of TMB oxide from ZnFe-LDH@Cu was recorded by UV-*vis* spectrometer (UV-N5000, China). Electron paramagnetic resonance spectroscopy (EMXmicro, Germany) was utilized to identify free radicals by electron spin resonance (ESR), with DMPO and TEMP used as trapping agents for active oxygen.

*X-ray absorption spectra (XAS) data analysis*

The XAS data, including XANES and EXAFS at the Cu K-edge of DT-ZnFe-LDH@Cu and reference samples, were conducted at the SSRF BL17B of the National facility for Protein Science in Shanghai (NFPS). The XANES data at the Cu K-edge were collected in transmission mode. The obtained XAS data were processed using Athena (version 0.9.26) for background, pre-edge line, and post-edge line calibrations. Fourier transformed fitting was then performed in Artemis (version 0.9.26). The fitting used k^3^ weighting, a k-range of 2.0-10.8 Å^-1^, and an R range of 1.8-2.8 Å. Four parameters were fitted: coordination number (C.N.), bond length (R), Debye-Waller factor (σ^2^), and E_0_ shift (ΔE_0_), without fixing, constraining, or correlating any of them. For Wavelet Transform analysis, the χ(k) exported from Athena was imported into the Hama Fortran code. The parameters were as follows: R range, 1-4 Å; k-range, 0-16 Å^–1^; k weight, 2; and the Morlet function with κ=10, σ=1 was used as the mother wavelet to provide the overall distribution.

*Determination of Singlet Oxygen by ABDA and DPBF Fluorescent Probes*

We combined ZnFe-LDH, Cu-SAzymes, ZnFe-LDH@Cu, and DT-ZnFe-LDH@Cu (100 μg/mL) with ABDA reagent in PBS (pH = 7.4) solution. Then, H_2_O_2_ (1 mM) was added to the dispersion. The ABDA reacted with ^1^O_2_, generating the corresponding internal peroxide, and its absorbance within the 300-400 nm was recorded by UV-*vis*.

ZnFe-LDH, Cu-SAzymes, ZnFe-LDH@Cu, and DT-ZnFe-LDH@Cu (100 μg/mL) were combined with DPBF in PBS (pH = 7.4) solution. Then, H_2_O_2_ (1 mM) was added to the dispersion. The DPBF reacted with ^1^O_2_ to form internal peroxides, and their absorbance at 410 nm was subsequently recorded by UV-*vis*.

*Antibacterial Assay of DT-ZnFe-LDH@Cu In Vitro*

1. Microbial Growth Curve Inhibition Test

DT@Cu, DT-ZnFe-LDH, and DT-ZnFe-LDH@Cu were mixed with suspensions of *P. aeruginosa,* MRSA, *S. aureus*, and *E. coli* (1×10^6^ CFU), respectively in LB medium. Next, the mixed suspension from the different treatment groups were subsequently transferred to a 96 -well plate and incubated at 37 °C incubator for 24 h (repeated 3 times for each group). OD_600_ values were then recorded at 0, 5, 10, 15, 20, and 24 h.

(2) Morphological Characterization of Bacteria

The bacterial morphology of DT@Cu, DT-ZnFe-LDH, and DT-ZnFe-LDH@Cu before and after incubation was recorded using SEM. Following the antibacterial assays, the mixture was centrifuged at 6000 r/min for 2 min and washed with cold PBS for 3 times to collect the bacteria. The bacterial pellets were then fixed in the dark at 4 °C with 2.5 % glutaraldehyde for 12 h. After fixation, the samples underwent ultrasonic washing with increasing concentrations of ethanol  (10, 30, 50, 70, 90, and 100 %) for 2 min, followed by centrifugation at 8000 rpm for 3 min. Finally, 5 μL fixed bacterial suspension drops were placed on a clean silicon wafer and allowed to dry naturally. Then, the conductive adhesive was coated with gold, and the morphology of the bacteria was characterized using SEM.

(3) Bacterial ROS Detection

The ROS produced by *P. aeruginosa* were detected using a DCFH-DA kit. To put it simply, 1×10^6^ CFU bacteria were incubated with various treatments, including PBS + H_2_O_2_, DT@Cu + H_2_O_2_, DT-ZnFe-LDH + H_2_O_2_, and DT-ZnFe-LDH@Cu + H_2_O_2_ (100 μg/mL) at 37 °C. Then, DCFH-DA (20 μM) fluorescent probe was added and the mixture was incubated for an additional 15 minutes. Fluorescence was observed with digital microscope, and the results were quantifiedwith Image-J software.

*In Vitro Biosafety Tests of DT-ZnFe-LDH@Cu*

Human corneal epithelial cells (HCECs) and mouse embryonic fibroblasts (NIH/3T3) cells were used to evaluate the cytotoxicity of DT-ZnFe-LDH@Cu. Different concentrations of DT-ZnFe-LDH@Cu (25, 50, 100, 150, and 200 μg/mL) were incubated with 3 mL Dulbecco Modified Eagle medium (DMEM/F-12) supplemented with 10 % fetal bovine serum (FBS, HyClone) and 1 % insulin-transferrin selenium (ITS-G) for 24 h. The leaching solution was filtered and sterilized using a filter, with fresh DMEM serving as the control group (n = 3). For the HCECs, cells were inoculated in 24-well plates with a density of 1×10^4^ cells per well and cultured in an incubator at 37 °C and 5 % CO_2_ for 24 h. On the second day, the cells were cultured for another 24 h with a different component leaching solution (500 μL/well) instead of the medium. Then, 100 μL 0.01 % fluorescein diacetate (FDA) solution was added to each well, which was rinsed twice with PBS after reaction for 5 min. The fluorescence images of cells were observed under inverted fluorescence microscope and photographed. The number of cells in morphology was calculated by Image-J software. NIH/3T3 cells were also inoculated into 24 -well plates of DMEM medium containing 10 % fetal bovine serum at a density of 1×10^4^ cells per well, and incubated in an incubator containing 5 % CO_2_ at 37 °C for 24 h to make the cells stick to the wall. Then, the extracts of different components of the material were added to the medium and cultured for 24 h, and the cell activity was observed by staining with 0.01 % fluorescein diacetate (FDA) solution. Then the number of cells in morphology was calculated by Image-J software.

*Hemolysis Assay in Vitro*

Fresh eyeball blood of healthy New Zealand white rabbits was collected, and red blood cells were isolated from serum by centrifugation (1500 rpm, 15 min). The red blood cells were washed 3 times with phosphate buffered saline (PBS) and diluted 10 times to create a red blood cell suspension (diluted to final concentration 5 %, v/v). The suspension of the test materials with different components was prepared. Positive control (+) and negative control (-) were prepared with deionized water and PBS, respectively. An equal volume ofthe red blood cell suspension was mixed with each sample solution, incubated at 37 °C for 30 min, and then centrifuged at 1500 rpm for 10 min. The supernatant was transferred to a 96 -well plate, and the absorbance was measured at 540 nm using a microplate meter. The hemolysis rate was calculated as follows:

$$hemolysis rate\left( \% \right)=\frac{{OD}_{sample}-{OD}_{PBS}}{{OD}_{water}-{OD}_{PBS}}\times100\%$$

*Statistical Analysis*

The experimental data in this study were statistically analyzed, and the relevant results were reported as mean ± standard deviation (n = 3), in the case of statistically significant differences, and the statistical analysis was implemented using the GraphPad Prism 9 software. Unlike comparing multiple groups by oneway variance analysis, the statistically significant differences between the two groups were compared by unpaired t-tests. P-values were calculated using ANOVA, and the levels of significance were labeled as non-significant (n.s.), *(P < 0.05), **(P < 0.01), or ***(P < 0.001).

References

[1] Z. Chen, P. Chen, Y. Zhu, J. Qian, X. Huang, W. Zhang, H. Zhang, Q. Mo, Y. Lu, Y. Zhang, *Adv. Funct. Mater.* **2023**, *33*, 2214693.

[2] P. Zhou, Y. Dai, X. Lin, Y. Song, Y. Pang, R. Chen, R. Xiao, *Adv. Funct. Mater.* **2024**, *34*, 2400875.

[3] W. Lu, Y. Guo, Y. Yue, J. Zhang, L. Fan, F. Li, Y. Zhao, C. Dong, S. Shuang, *Chem. Eng. J.* **2023**, *468*, 143615.

[4] M. Feng, Q. Zhang, X. Chen, D. Deng, X. Xie, X. Yang, *Biosens. Bioelectron.* **2022**, *210*, 114294.

[5] C. Ma, Y. Xu, L. Wu, Q. Wang, J. Zheng, G. Ren, X. Wang, X. Gao, M. Zhou, M. Wang, H. Wei, *Angew. Chem. Int. Ed.* **2022**, *61*, 25.

[6] L. Chen, C. Ding, K. Chai, B. Yang, W. Chen, J. Zeng, W. Xu, Y. Huang, *ACS Nano* **2023**, *17*, 18148-18163.

[7] X. Wang, W. Hu, X. Xia, C Wang, *Adv. Funct. Mater.* **2023**, *33*, 10.

[8] M. Tang, Y. Shi, L. Lu, J. Li, Z. Zhang, J. Ni, W. Wang, Y. Zhang, T. Sun, Z. Wu, *Chem. Eng. J.* **2022**, *449*, 137847.

[9] S. Dong, Y. Dong, B. Liu, J. Liu, S. Liu, Z. Zhao, W. Li, B. Tian, R. Zhao, F. He, S. Gai, Y. Xie, P. Yang, Y. Zhao, *Adv. Mater.* **2022**, *34*, 7.

[10] J. Liu, S. Dong, S. Gai, Y. Dong, B. Liu, Z. Zhao, Y. Xie, L. Feng, P. Yang, J. Lin, *ACS Nano* **2023**, *17*, 20402-20423.

[11] Y. Zuo, D. Zhong, C. Huang, X. Ding, K. Qu, X. Wang, Y. Xu, *ACS Appl. Nano Mater.* **2023**, *6*, 3357-3366.

[12] Q. Qiao, J. Wang, K. Long, L. Li, J. Chen, Y. Guo, Z. Xu, Y. Kuang, T. Ji, C. Li, *Nano Today* **2024**, *54*, 102059.

[13] W. Zhen, Y. Liu, W. Wang, M. Zhang, W. Hu, X. Jia, C. Wang, X. Jiang, *Angew. Chem. Int. Ed.* **2020**, *59*, 9491.

[14] S. Liu, Q. Bai, Y. Jiang, Y. Gao, Z. Chen, L. Shang, S. Zhang, L. Yu, D. Yang, N. Sui, Z. Hu, *Small* **2024**, *20*, 2308403.

[15] Y. Wang, V.K. Paidi, W. Wang, Y. Wang, G. Jia, T. Yan, X. Cui, S. Cai, J. Zhao, K. Lee, L. Lee, K. Wong, *Nat. Commun.* **2024**, *15*, 2239.

[16] M. Wang, X. Zhou, Y. Li, Y. Dong, J. Meng, S. Zhang, L. Xia, Z. He, L. Ren, Z. Chen, X. Zhang, *Bioact. Mater.* **2022**, *17*, 289-299.

[17] K. Kim, J. Lee, O.K. Park, J. Kim, J. Kim, D. Lee, V.K. Paidi, E. Jung, H.S. Lee, B. Lee, C.W. Lee, W. Ko, K. Lee, Y. Jung, C. Lee, N. Lee, S. Back, S.H. Choi, T. Hyeon, *Adv. Mater.* **2023**, *35*, 2207666.

[18] M. Wang, C. Yang, M. Chang, Y. Xie, G. Zhu, Y. Qian, P. Zheng, Q. Sun, J. Lin, C. Li, *Nano Today* **2023**, *52*, 101981.

[19] Z. Lin, T. Dong, L. Niu, X. Zhang, M. Wang, X. Liu, Y. Cai, A. Liu, *Chem. Eng. J.* **2024**, *482*, 148775.

[20] K. Li, Y. Miao, K. Song, S. He, G. Zhang, G. Waterhouse, S. Guan, S. Zhou, *Chem. Eng. J.* **2023**, *471*, 144693.

[21] Y. Rao, G. Xu, Z. Zhang, W. Wang, C. Zhang, M. Zhao, Y. Qu, W. Li, M. Ji, Y. Liu, Y. Li, *Chem. Eng. J.* **2023**, *465*, 142961.


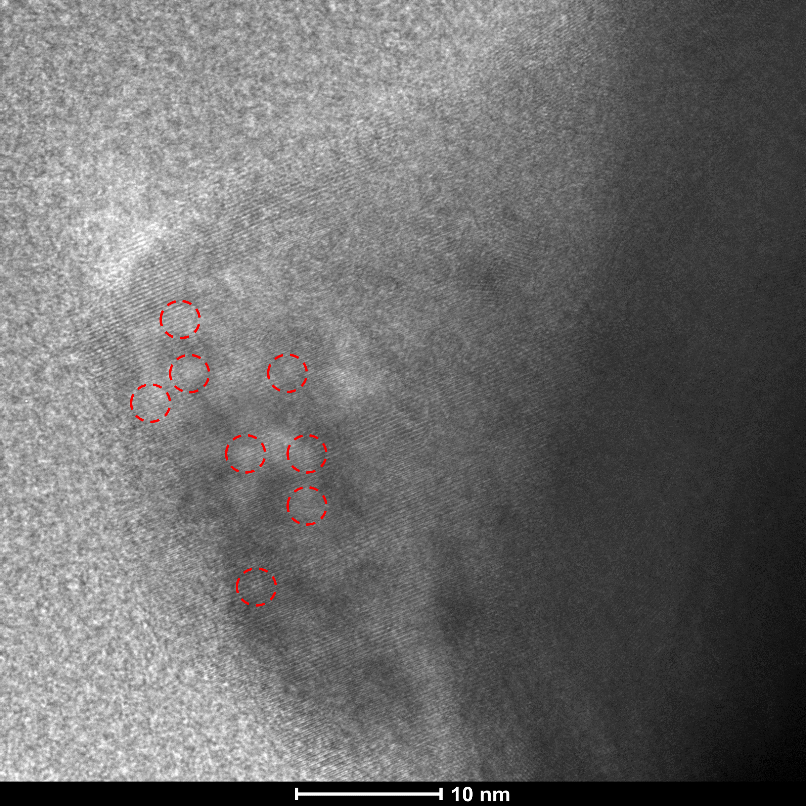


**Figure S1.** Ultrahigh-resolution field emission transmission electron microscopy images of Cu-SAzymes.


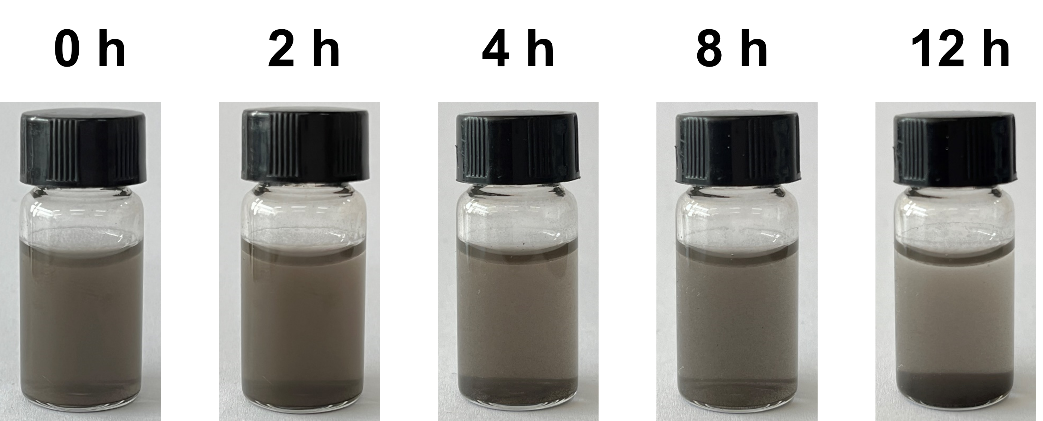


**Figure S2.** Dispersibility of ZnFe-LDH@Cu at the concentration of 100 μg/mL in ultrapure water.


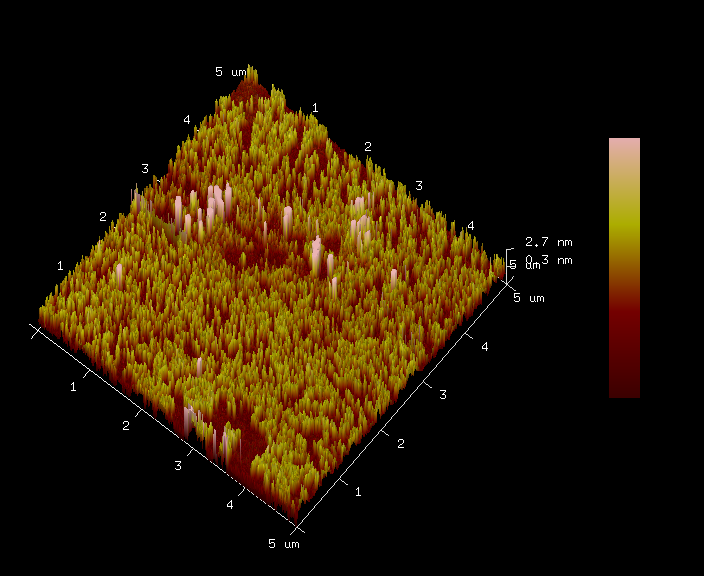


**Figure S3.** Three-dimensional AFM images of ZnFe-LDH@Cu.


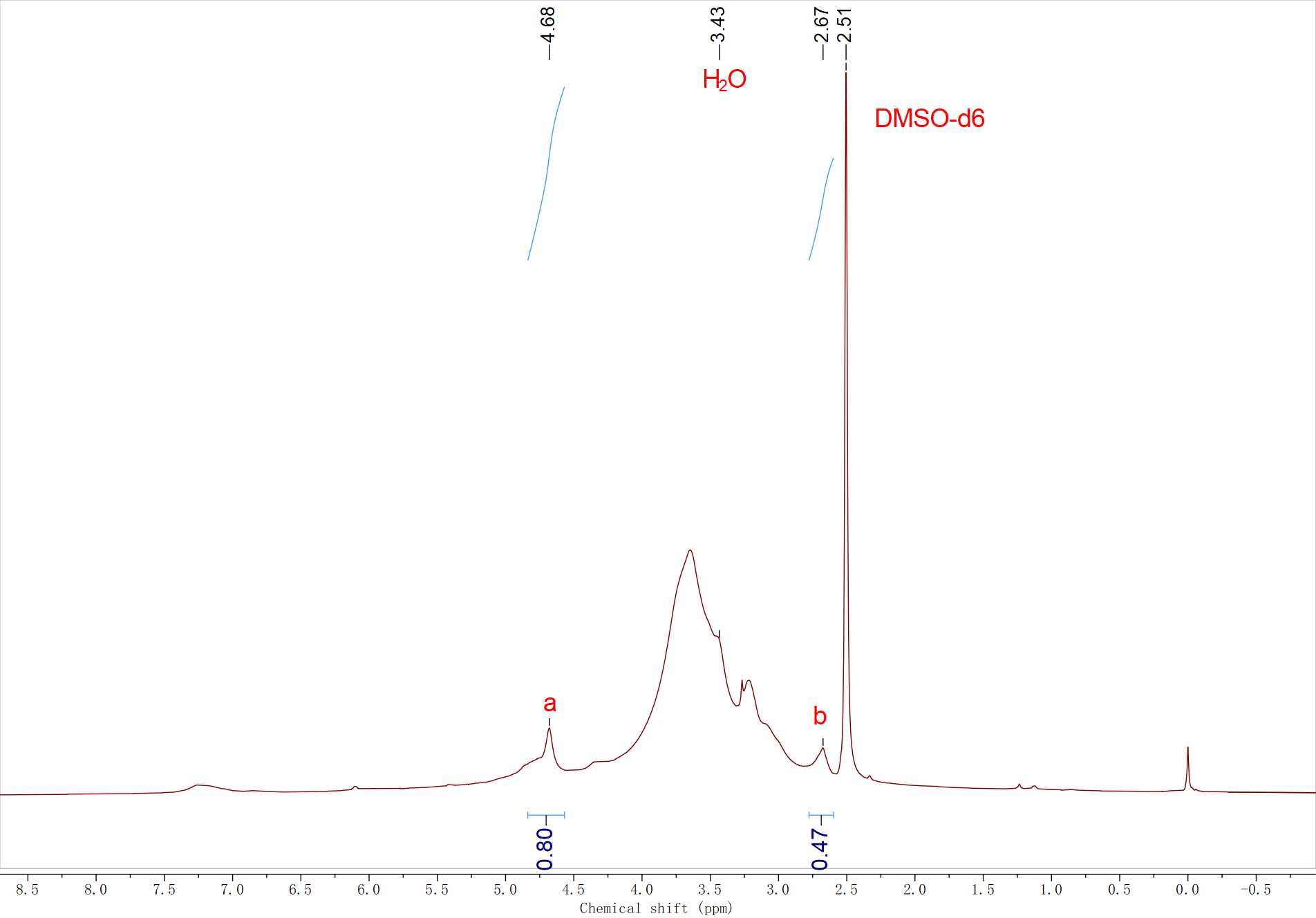


**Figure S4.** ^1^H NMR spectrum of Dex-NH_2_.


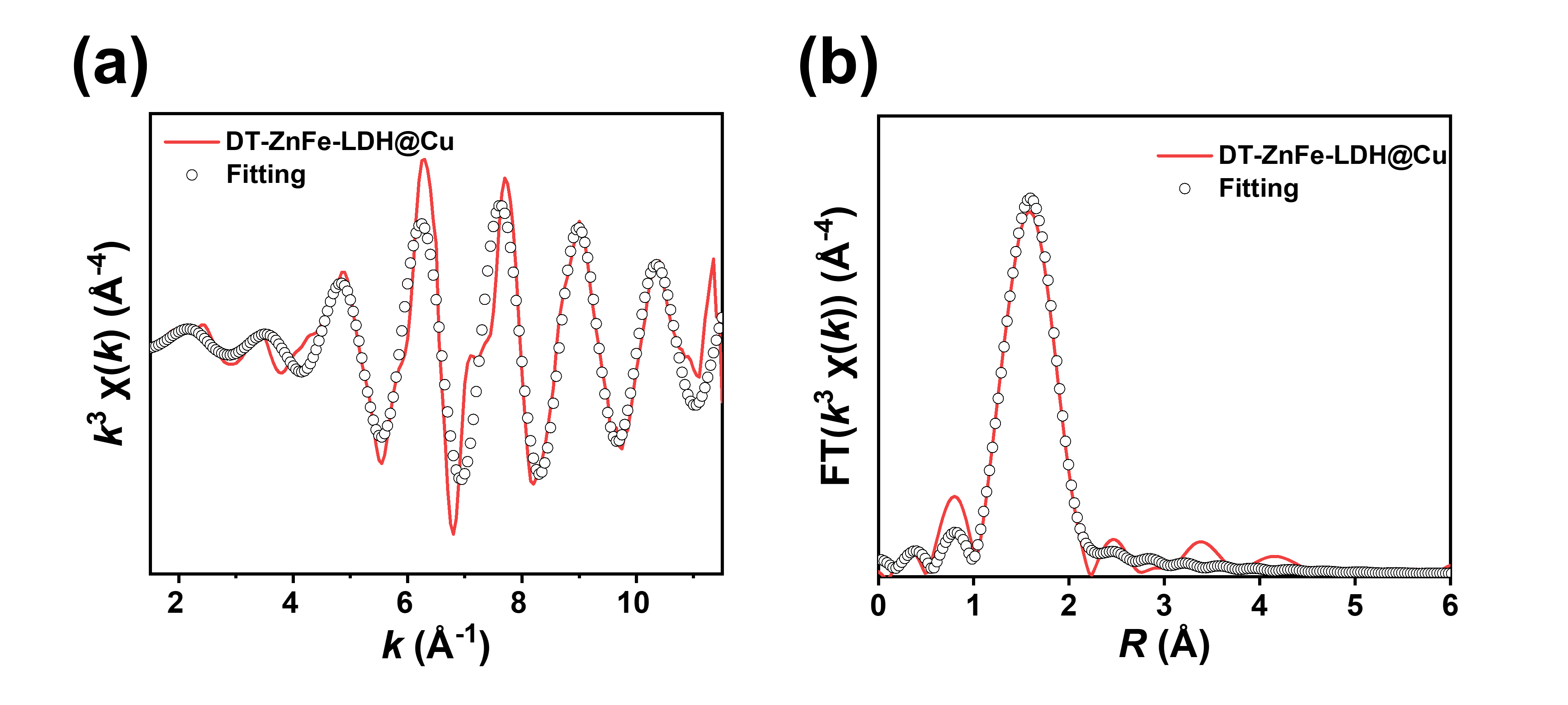


**Figure S5.** (a) Corresponding EXAFS fitting curves of DT-ZnFe-LDH@Cu at k space. (b) Corresponding EXAFS fitting curves of DT-ZnFe-LDH@Cu at R space.


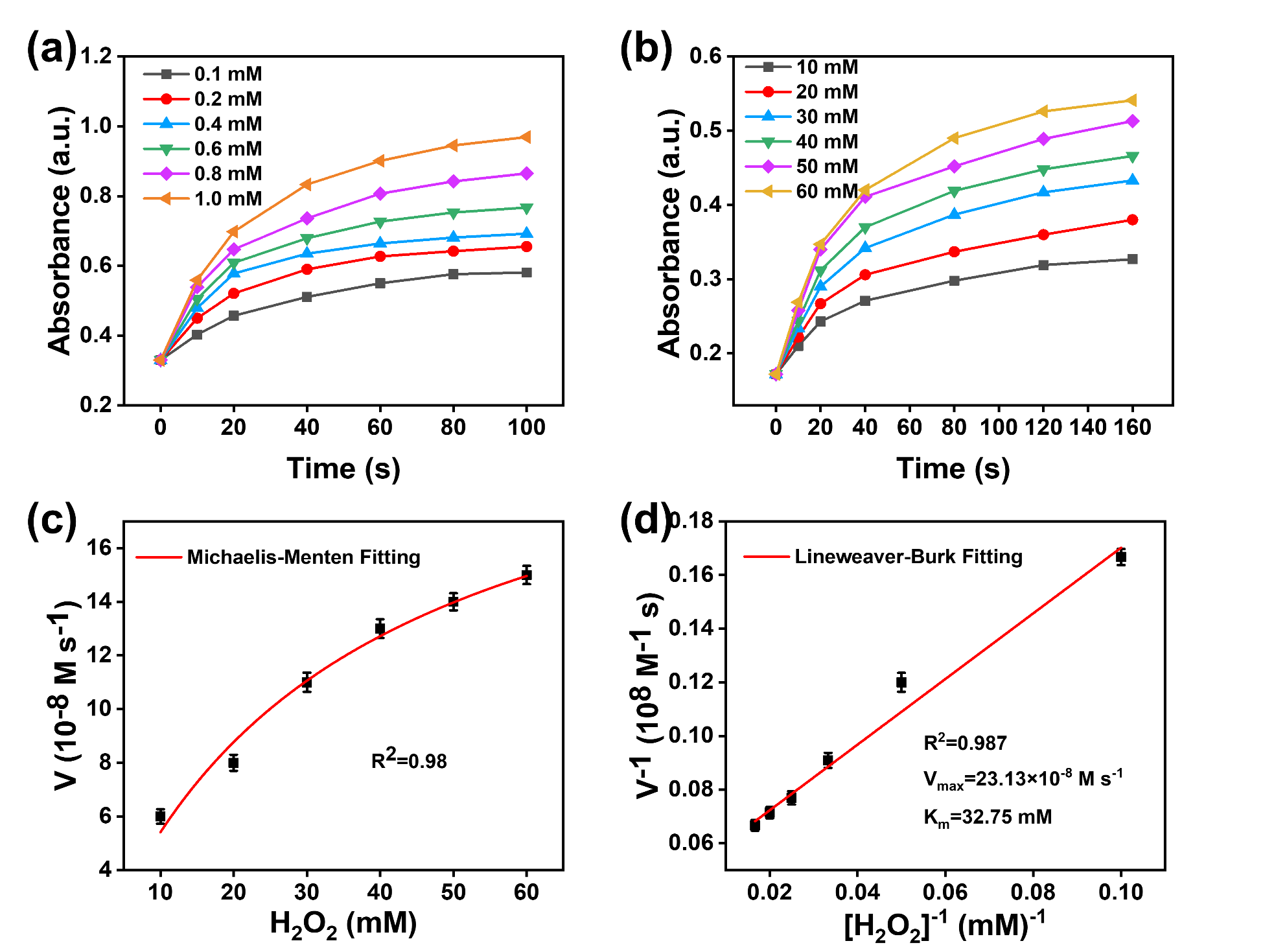


**Figure S6.** (a) Effect of different concentrations of TMB on the POD-like activity of DT-ZnFe-LDH@Cu. (b) Effect of different concentrations of H_2_O_2_ on the POD-like activity of DT-ZnFe-LDH@Cu. (c) Michaelis–Menten kinetic analysis for DT-ZnFe-LDH@Cu. (d) Lineweaver–Burk plot of DT-ZnFe-LDH@Cu.


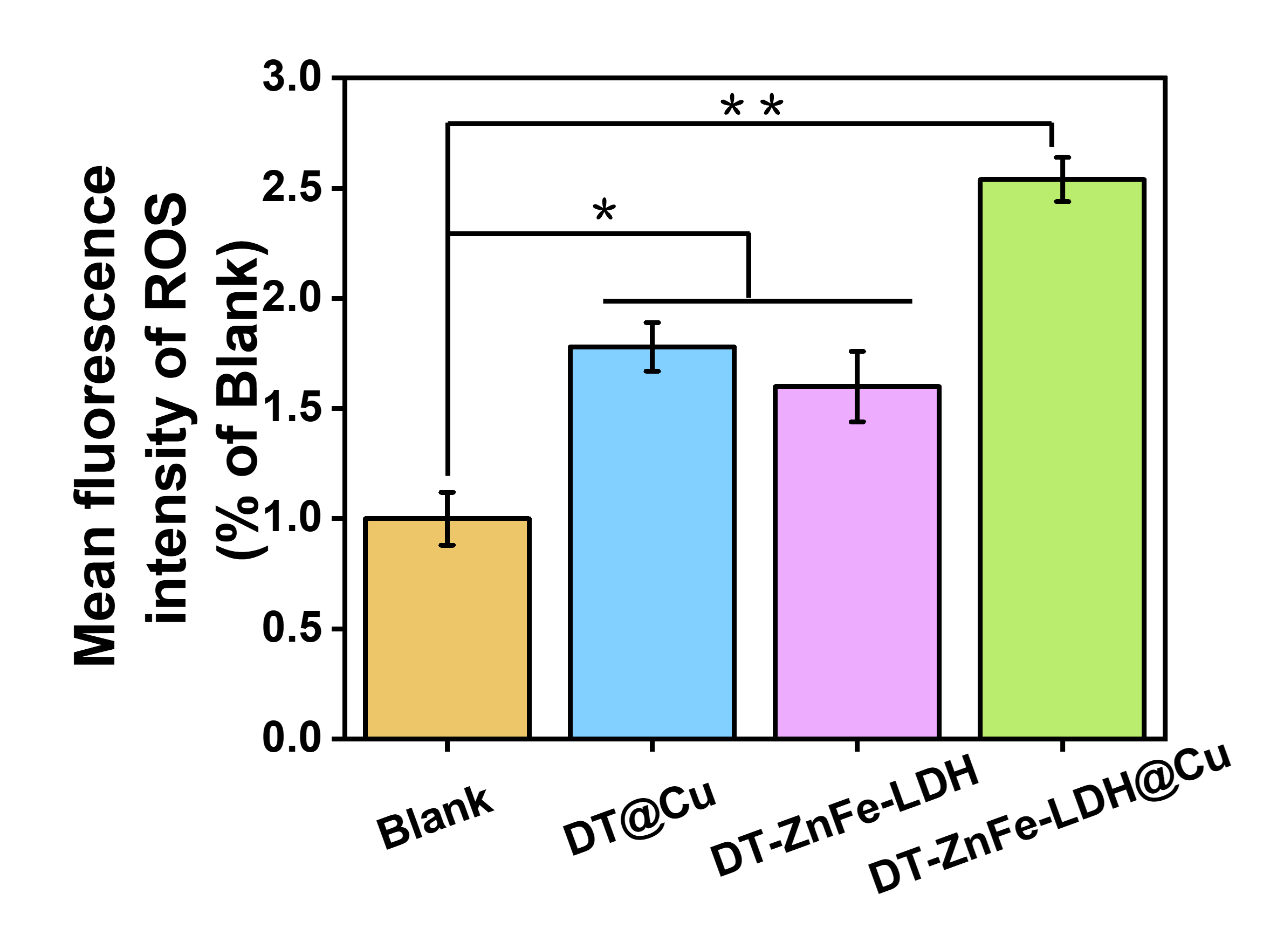


**Figure S7.** The ROS levels in *P. aeruginosa* were quantified by DCFH-DA fluorescent probe in Figure 4j. Data are presented as mean ± SD (n = 3). (*p < 0.05 and **p < 0.01).

**
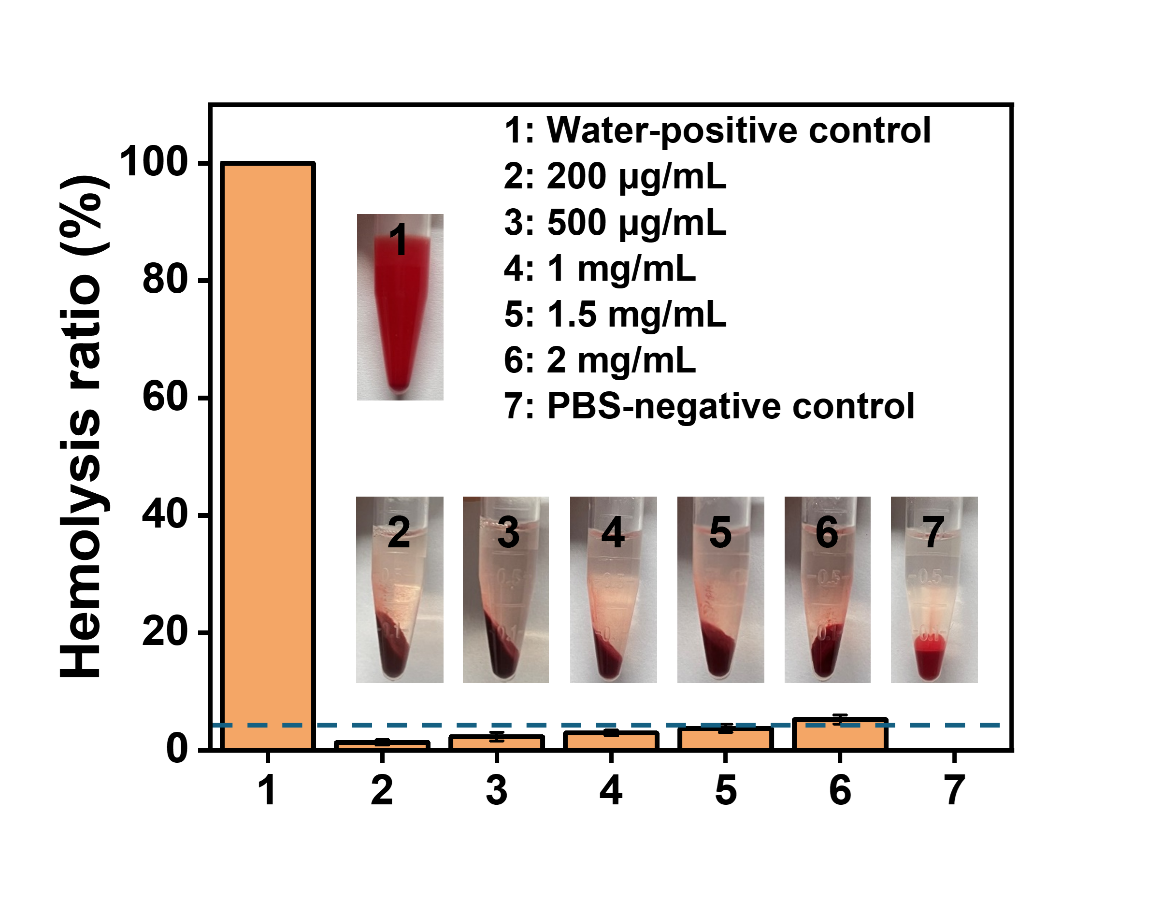
**

**Figure S8.** Erythrocyte hemolysis test at different concentrations of DT-ZnFe-LDH@Cu.

**
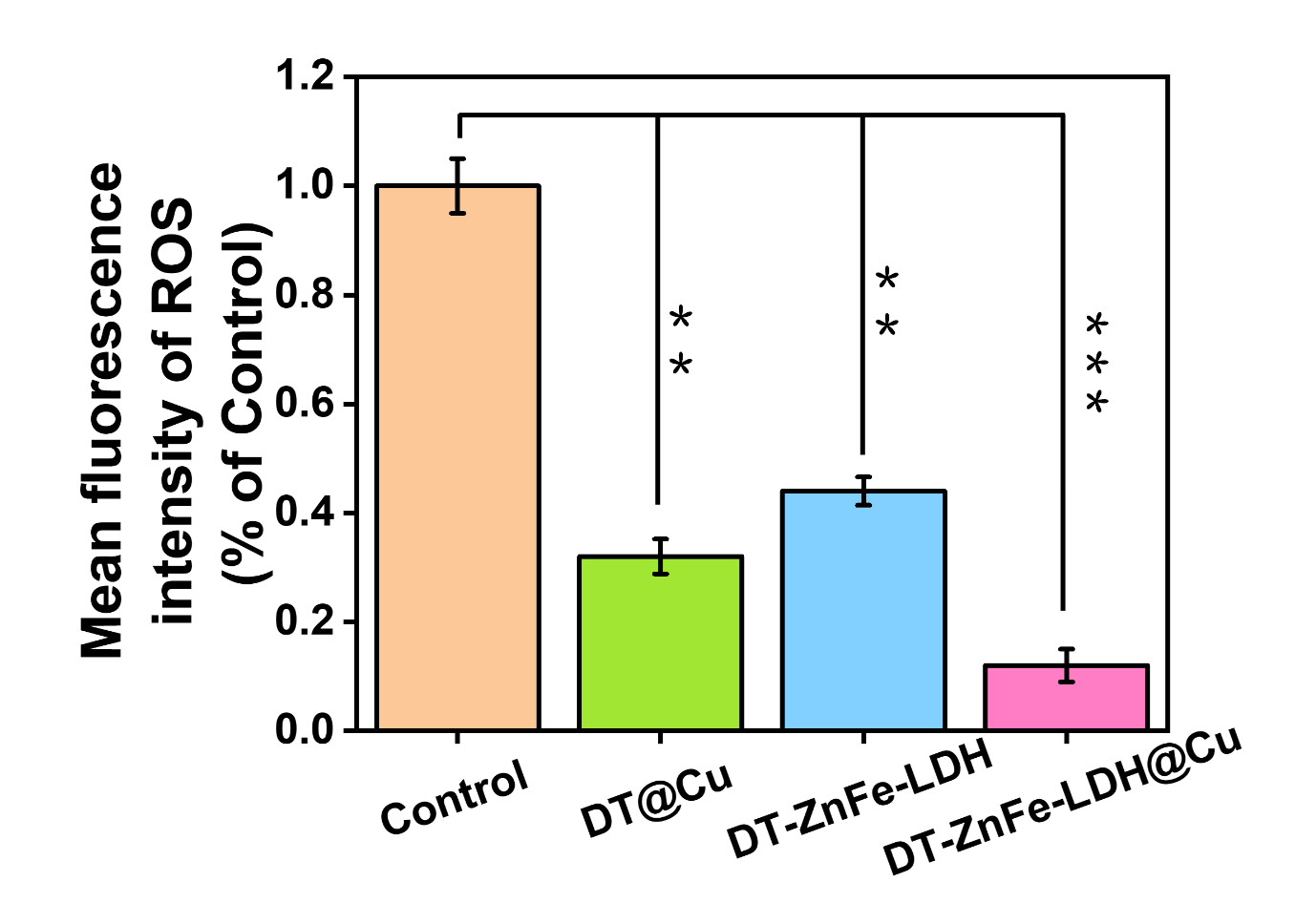
**

**Figure S9.** Intracellular ROS levels were quantified by DCFH-DA fluorescent probe in Figure 5f. Data are presented as mean ± SD (n = 3). (**p < 0.01 and ***p < 0.001.)

**Table S1.** Percentage composition of elements in ZnFe-LDH@Cu.

| **Element** | **wt %** | **n (Zn/Fe/Cu)** |
| --- | --- | --- |
| **Zn** | 23.75 ± 0.01 | 3.37:3.46:1 |
| **Fe** | 24.41 ± 0.05 |  |
| **Cu** | 7.05 ± 0.02 |  |

**Table S2.** EXAFS fitting parameters at the Cu K-edge for various samples (*S_0_^2^*=0.97).

| **Sample** | **Path** | ***C.N.^a^*** | ***R^b^(Å)*** | **σ^2c^×10^3^(Å^2^)** | **ΔE^d^(eV)** | ***R* factor^e^** |
| --- | --- | --- | --- | --- | --- | --- |
| DT-ZnFe-LDH@Cu | Cu-N | 3.66±0.83 | 1.94 | 9.0 | 4.82±0.98 | 0.0038 |

^a^ Coordination numbers. ^b^ Bond distance. ^c^ Debye-Waller factors. ^d^ Inner potential correction. ^e^ Goodness of fit. *Ѕ*_0_^2^ was set to 0.97. The data range used for data fitting in k-space (∆k) and R-space (∆R) are 2-10.8 Å^-1^ and 1.8-2.8 Å, respectively.

**Table S3.** Kinetic parameters of reported nanozymes with POD-like activity.

| **Catalyst** | **Substrate** | ***V*_max_ (10^–8^ M·s^–1^)** | ***K*_m_ (mM)** | **Reference** |
| --- | --- | --- | --- | --- |
| ZnFe-LDH@Cu | H_2_O_2_ | 23.13 | 32.75 | **This work** |
|  | TMB | 51.40 | 0.22 |  |
| Co NSs | H_2_O_2_ | 0.96 | 63.74 | [1] |
|  | TMB | 1.60 | 1.26 |  |
| Fe_3_O_4_@COF@Os | H_2_O_2_ | 110 | 109 | [2] |
|  | TMB | 134 | 1.12 |  |
| Mo-CDs | H_2_O_2_ | 4.76 | 0.18 | [3] |
|  | TMB | 3.20 | 0.25 |  |
| ZnBNC | H_2_O_2_ | 11.24 | 0.11 | [4] |
|  | TMB | 10.86 | 1.24 |  |
| Zn/Mo DSAC-SMA | H_2_O_2_ | 33.33 | 40.32 | [5] |
|  | TMB | 3.84 | 0.43 |  |
| n-MoSe_2_ | H_2_O_2_ | 56.62 | 2.29 | [6] |
|  | TMB | 18.34 | 0.25 |  |
| SZN-MOFs | H_2_O_2_ | 30 | 132.35 | [7] |
|  | TMB | 14.60 | 0.039 |  |

**Table S4.** Kinetic parameters of reported nanozymes with CAT-like activity.

| **Catalyst** | **Substrate** | ***V*_max_ (mg·L^–1^·min^–1^)** | ***K*_m_ (mM)** | **Reference** |
| --- | --- | --- | --- | --- |
| ZnFe-LDH@Cu | H_2_O_2_ | 6.01 | 14.87 | **This work** |
| Ti_3_C_2_/CeO_2_-PVP | H_2_O_2_ | 0.47 | 57 | [8] |
| CeO_2_ | H_2_O_2_ | 0.34 | 77.64 | [9] |
| AuCuPt-PpIX | H_2_O_2_ | 6.5 | 70.73 | [10] |
| CoSe_2_ | H_2_O_2_ | 1.307 | 2.97 | [11] |
| Ti_3_C_2_Tx | H_2_O_2_ | 0.02 | 85.58 | [12] |
| IrOx NPs | H_2_O_2_ | 10.83 | 187.95 | [13] |
| MnOx/GDY | H_2_O_2_ | 6.37 | 1.10 | [14] |

**Table S5.** Kinetic parameters of reported nanozymes with OXD-like activity.

| **Catalyst** | **Substrate** | ***V*_max_ (10^–8^ M·s^–1^)** | ***K*_m_ (mM)** | **Reference** |
| --- | --- | --- | --- | --- |
| ZnFe-LDH@Cu | TMB | 5.37 | 0.37 | **This work** |
| FePc@2D-Cu–N–C | TMB | 89.13 | 0.60 | [15] |
| ZIF8/Au NPs | TMB | 2.83 | 14.21 | [16] |
| FeNC-edge | TMB | 4.22 | 0.37 | [17] |
| Fe SAzyme | TMB | 2.86 | 0.11 | [18] |
| Co_3_O_4_ NWs | TMB | 11.20 | 0.067 | [19] |
| CuMn-DAzymes | TMB | 8.32 | 0.59 | [20] |
| Pt-AuNS | TMB | 0.42 | 4.53 | [21] |

**Table S6.** Primer sequences for RT-qPCR.

| **Gene** | **Forward primer (5´-3´)** | **Reverse primer (5´-3´)** |
| --- | --- | --- |
| GAPDH | AGGTGGTGAAGCAGGCATCT | GGCATCGAAGGTGGAAGAGT |
| NF-κB | CTCTGGCACAGAAGTTGGGT | TCCCGGAGTTCATCTCATAGT |
| IL-6 | ATAGTCCTTCCTACCCCAATTTCC | GATGAATTGGATGGTCTTGGTCC |
